# Local Ecological Knowledge Insights Into the Distribution and Activity Patterns of Temminck's Pangolin in Ruaha Landscape, Tanzania

**DOI:** 10.1002/ece3.71987

**Published:** 2025-08-16

**Authors:** Rose Peter Kicheleri, Courtney Hughes, Michael Honorati Kimaro, Charles Peter Mgeni, Nyemo Amos Chilagane, Hillary Thomas Mrosso, Simon Joshua Chidodo, Fenrick Filbert Msigwa, Elisante Azaeli Kimambo, Rajabu Joseph Kangile, George Bunyata Bulenga, Camille Warbington

**Affiliations:** ^1^ Tanzania Research and Conservation Organization Morogoro Tanzania; ^2^ Sokoine University of Agriculture Morogoro Tanzania; ^3^ GELIFES Institute University of Groningen Groningen the Netherlands

**Keywords:** community, conservation, distribution, local knowledge, planning, Tanzania

## Abstract

Tanzania is home to three species of pangolins: Temminck's pangolin (*Smutsia temminckii*), giant ground pangolin (*Smutsia gigantea*), and white‐bellied pangolin (*Phataginus tricuspis*). However, distribution and habitat preferences have yet to be well known across the Ruaha landscape, encompassing the core of Ruaha National Park and adjacent protected and unprotected village lands. This area is thought to hold Temminck's pangolin. Drawing upon local knowledge to help inform conservation planning, we used semi‐structured interviews among village members to investigate the distribution and activity pattern of Temminck's pangolins in the Ruaha landscape. Our results show that village lands hold potential habitats for pangolins, and unsurprisingly, that human land use by activity type and human behavior itself influences pangolin observations across the landscape, more so than pangolin ecology. We also learned that more than half of our study's participants did not perceive a decreasing population trend in pangolins over 5 years, despite reports from authorities. Our study provides novel and important baseline information about the distribution of pangolins in the Ruaha landscape, which can be used for spatially relevant conservation planning at local and national scales. Given their willingness to share local knowledge about pangolins and participate in pangolin conservation, we suggest that village members be actively engaged in pangolin conservation efforts, including training on monitoring and reporting pangolin population and distribution, and assisting in habitat management.

## Graphical abstract

Ruaha landscape in Tanzania is home to Temminck's ground pangolin (*Smutsia temminckii*). However, their population, distribution, and habitat preferences have yet to be well known despite pangolins being targeted for illegal trafficking to international markets. This paper used indigenous knowledge to understand human–pangolin interactions in this landscape.

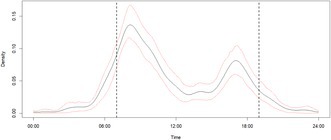

## 1 Introduction

As one of the most trafficked animals in the world, pangolins are listed as either vulnerable, endangered, or critically endangered across their global range (Pietersen, Jansen, and Connelly 2019; Pietersen et al. 2019). Widespread poaching, illegal trafficking, and habitat loss are largely contributing factors to population decline (Pietersen, Jansen, and Connelly 2019; Pietersen et al. 2019). Pangolins are poached and trafficked for use as a food resource, as well as for various local and international traditional and cultural practices (Baiyewu et al. 2018; Willcox et al. 2019). Even though pangolin populations are known to be in peril, significant knowledge gaps remain, particularly for African species (Heighton and Gaubert 2021).

Three pangolin species are found in Tanzania, although the distribution of each species is based on incidental observations (Foley et al. 2014). Temminck's pangolin (*Smutsia temminckii*) is believed to be widespread across various ecosystems in Tanzania. In contrast, the white‐bellied (or tree) pangolin (*Phataginus tricuspis*) and giant pangolin (*Smutsia gigantea*) occur in the northwestern region of the country (Foley et al. 2014; Nixon et al. 2019; Pietersen, Jansen, and Connelly 2019; Pietersen et al. 2019). While Tanzania's Wildlife Act (URT 2009) and international treaties such as CITES Appendix I are intended to help prevent the trade of pangolins and their derivatives (Mrosso et al. 2022a, 2022b), the Tanzania Wildlife Management Authority (TAWA) and other research report an increase in seizures of pangolin scales and live pangolins country‐wide (Hariohay et al. 2023). Other threats that may affect the pangolin population in Tanzania include wildfire and human‐caused fires on croplands, expansion of crop cultivation, and the use of livestock guarding dogs, which may attack and injure or kill pangolins (Traoré and Lepage 2008; Katuwal et al. 2017).

**FIGURE 1:**
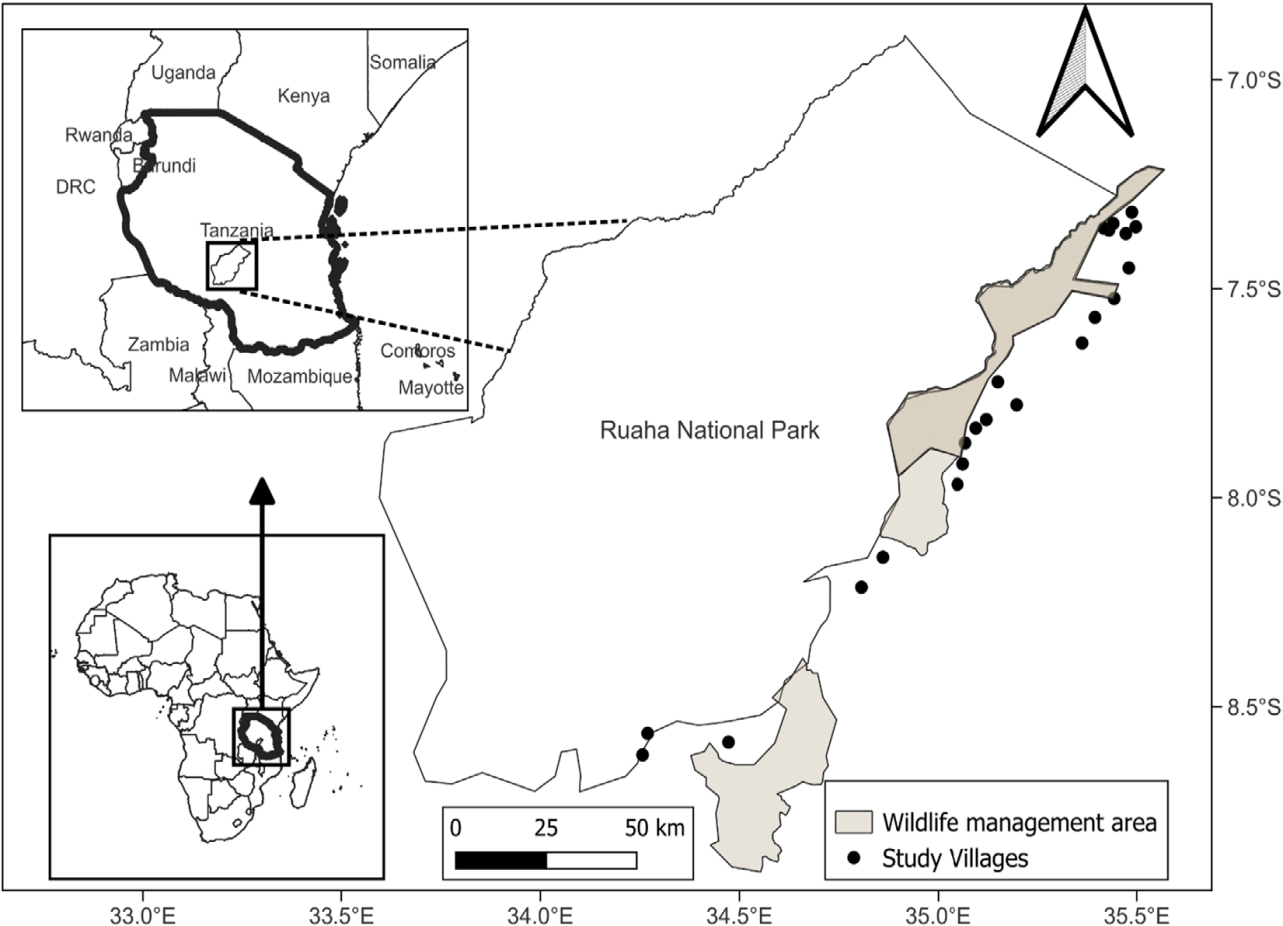
Map showing study villages adjacent to the Ruaha National Park (*Source:* Mgeni, Kicheleri et al. 2024).

Despite increasing awareness that pangolins are the most trafficked animal in the world (Heighton and Gaubert 2021), there is a lack of information on Tanzania's pangolin population and distribution, including for Temminck's pangolins in the Ruaha–Rungwa ecosystem (Willcox et al. 2019; Figure 1). This consists of a lack of information on local people's knowledge about and uses of this pangolin species, despite this data being crucial to developing contextually relevant conservation solutions (Fopa et al. 2020; Suwal et al. 2022). Indeed, engaging local village members to share their knowledge can be an essential data collection technique in conservation science, particularly for elusive species such as pangolins (Fopa et al. 2020). Moreover, providing opportunities for local people to get involved in conservation efforts can help recruit pangolin champions and, in turn, support monitoring and reporting on the pangolin population and distribution.

As part of a broader research project, we engaged the local villages adjacent to Tanzania's Ruaha National Park to share their local knowledge on pangolins across their village lands (Camino et al. 2020; Dawson et al. 2021). Our objectives were to understand the local pangolin spatial distribution, activity patterns, habitat associations, and the anthropogenic factors that may influence pangolin presence in the area. Our results will help provide important information to develop local and national‐level conservation action for pangolins, including an action plan, future monitoring and reporting, and community‐based conservation programs (Suwal et al. 2022).

## 2 Materials and Methods

### 2.1 Study Area

Ruaha National Park in south‐central Tanzania is surrounded by game reserves, game‐controlled areas, Wildlife Management Areas (WMAs), and village lands. Our study focused on the villages adjacent to the southern boundary of Ruaha National Park (Figure 1). This area is semi‐arid to arid. It is characterized by two dry seasons, from February to March, and from June to November (driest months), as well as two wet seasons, from December to January (least rainy months), and March to May (wettest months), while June to July are the coldest months (Abade et al. 2014; George and Kangalawe 2024). The landscape includes a diverse array of wildlife species, including Temminck's pangolin (Cusack et al. 2015).

The major ethnic groups residing within the area include small‐scale crop farmers from the Hehe, Bena, and Gogo tribes (Kimaro and Hughes 2023) and livestock keepers from the Barabaig, Maasai, and Sukuma tribes (Mrosso et al. 2022a, 2022b). In the Ruaha Valley, recorded uses of Temminck's pangolin reportedly vary by tribe, but generally include using body parts as good luck charms, as part of “medicinal” treatments for pneumonia, or live animals as part of rituals to forecast the future (Walsh 2007).

### 2.2 Study Design and Data Collection

We used semi‐structured interviews as a culturally appropriate, cost‐effective, and time‐efficient technique to collect data from a random selection of 21 different villages (*N* = 36) found adjacent to Ruaha National Park (Lavrakas 2008; Creswell and Creswell 2023). We selected the villages using Excel's random generator (Microsoft Corporation 2021) and then contacted each of the villages' leaders, requesting they develop a list of different individuals in their community they knew to be (a) engaged in wildlife hunting; (b) had reported a pangolin sighting/encounter to officials; and/or (c) were traditional healers (i.e., purposeful sample; Patton 2002; Rust et al. 2017). We invited these individuals to participate in face‐to‐face interviews. We developed our interview guide based on reviewing other similar studies (Trageser et al. 2017; Perera and Karawita 2020) and used a culturally appropriate and logistically feasible approach to gather data and engage with village members in applied research (Hughes et al. 2020; Kimaro and Hughes 2023). This approach helped us build rapport, awareness, and understanding of conservation research and researchers and increase the opportunity for collecting robust data and developing successful conservation programs (Hallwass et al. 2020; Willcox et al. 2019; Camino et al. 2020). We do note that qualitative techniques, as with other research involving human subjects, may have limitations and bias, including social desirability (Bergen and Labonté 2020).

Before beginning each interview, we read a description of the study scope to each participant and requested they provide verbal free and informed consent to proceed (SUA 2019; Brittain et al. 2020). Upon the culmination of each interview, we used chain referral to identify other additional village members to participate in our study, including crop farmers, livestock keepers, forest product gatherers, and business owners (Table S1; Penrod et al. 2003; Potgieter et al. 2017; D'Cruze et al. 2018; Hughes and Nielsen 2019). All responses were entered onto tablets by the research team using the Open Data Kit (ODK) application, and the interview transcripts were stored on the password‐protected laptop.

We collected demographic information from each participant, including their age, occupation, annual income, years of residence, self‐identified ethnic group, and level of education. For participants who reported having directly encountered a pangolin, we collected information on the time of day, type of habitat, land use category where the encounter occurred, how many pangolins were encountered, and what the respondent was doing when the encounter occurred (human activity). Land use categories were National Parks, Wildlife Management Areas (WMA) managed by villagers, and Village Land, where human settlement, crop cultivation, and livestock grazing are allowed. Some villages are located within the park and have a government registration number; therefore, we treated their boundary as village land. Human activity categories included Patrol and Hunting, Opportunistic Observation, Medicine Gathering, and Livelihood Activities (including house construction, crop farming, firewood collection, livestock grazing, and honey collection). Human activity categories were developed by the research team, based on local expert knowledge and prior testing of the interview guide. We also asked participants who reported a pangolin encounter how often they visited the habitat type where they encountered the pangolin, how often they encountered pangolins, and their perceived trend for the frequency of encountering pangolins. Finally, we asked participants about the trend they perceived to occur in encountering pangolins over 5 years; responses were limited to increasing, decreasing, no change, and uncertainty.

### 2.3 Data Analysis

All analyses were conducted using R software (R Core Team 2021). We used chi‐squared tests to assess the difference in frequency of responses about pangolin encounters and (1) land management type, (2) human activity, (3) habitat type where the encounter occurred, and (4) ethnic group. We corrected for variation in the number of survey respondents per ethnic group by testing for differences in the proportion of respondents of each ethnic group reporting pangolin encounters. We combined ethnic groups with *n* ≤ 5 into a single group for the chi‐squared test. We also used a chi‐squared test to assess the perceived trend in encountering pangolins over the past 5 years. For chi‐squared and other analyses, we considered *p* < 0.05 as an indication of a statistically significant difference.

Logistic regression was used to test the association of demographic factors with pangolins encountered. Respondents who observed pangolins were coded as “1” and those who did not observe pangolins were coded as “0”; then, 1 or 0 was treated as a response variable. To select variables to include in the regression, we first checked for correlations between our predictor variables of gender, age, occupation, income, length of residence, and education. If variables were identified as strongly correlated (*R* > 0.8), we excluded one of the correlated variables from the regression. We fit the full model and interpreted the *p‐*values and coefficients to assess which demographic variables have the strongest relationship to pangolin encounters, given the effect of other demographic factors. Gender, occupation, and education level were treated as factors in the regression analysis. We combined the 12 ethnic groups from our total number of respondents, given that some ethnic groups had *n* ≤ 5, to facilitate chi‐square tests.

We also used quasi‐Poisson regression analysis to assess the observed variation of the reported pangolin group size, relative to different human activity and land use categories. As the response variable, the number of pangolins observed is a count variable. We treated human activity and land use categories as factors in the regression. We used the R package “activity” to assess the activity patterns of pangolins encountered over the 24‐h cycle.

We interviewed a total of 386 participants from 21 different villages adjacent to the Ruaha National Park's boundary. Participants originated from 19 different tribes, of which approximately 52% (*n* = 199) were from the Hehe tribe (Table S1). About 93% (*n* = 356) of participants were males, which is common given the criteria of our study (i.e., hunters, crop farmers, healers, etc.) and the common culture of patriarchy. The average age of participants was 54 years, ranging from 19 to 102 years. Seventy‐seven percent of participants (*n* = 298) indicated their primary livelihood is crop cultivation, and 69% (*n* = 265) had a primary education level.

## 3 Results

### 3.1 Pangolin Observations Relative to Land Use, Human Activity, and Habitat Type

**FIGURE 2:**
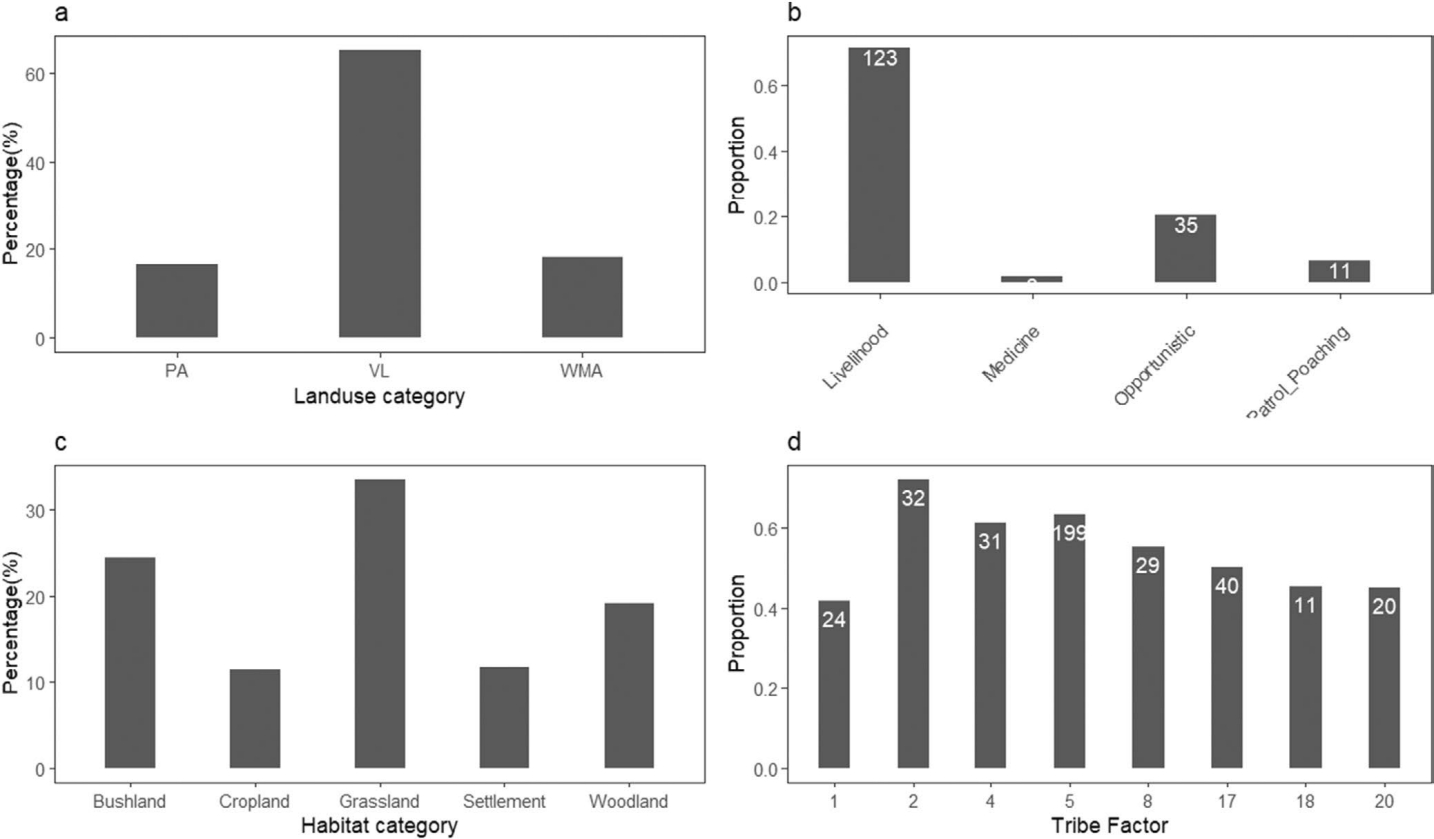
Pangolin sightings in different (a) land use types, (b) human activity categories, (c) habitat types, and (d) proportion of survey respondents reporting pangolin sightings by ethnic group. NP indicates national park, VL indicates village land, and WMA indicates wildlife management areas managed by local communities. Tribe Factor refers to the ethnic group of respondents, where ethnic groups with *n* > 5 are grouped (Tribe Factor 20). The number above or within the bars is the total number of reports attributed to the given category.

Pangolin observations varied significantly with land use type (*χ* ^2^ = 45.14, df = 2, *p* < 0.001, Figure 2a), human activity (*χ* ^2^ = 211.35, df = 3, *p* < 0.001, Figure 2b), and habitat type (*ꭓ* ^2^ = 16.707, df = 4, *p* = 0.002, Figure 2c). Approximately 65% of pangolin observations occurred in the village land compared to inside the park and wildlife management areas (Figure 2a). Respondents (*n* = 169) reported that human activities associated with high pangolin observations included Livelihood Activities, which consisted of collecting materials (i.e., wood) for house construction, firewood collection, and conducting crop farming, livestock grazing, and honey collection (Figure 2b). The habitat types with the highest proportion of reported pangolin observations were Grassland, Bushland, and Woodland compared to Settlement areas and Cropland (Figure 2c).

### 3.2 Socio‐Demographic Factors Influencing Pangolin Observations

We found no difference in the proportion of people reporting a pangolin encounter across ethnic groups (*ꭓ* ^2^ = 10.748, df = 7, *p* = 0.1, Figure 2d).

**FIGURE 3:**
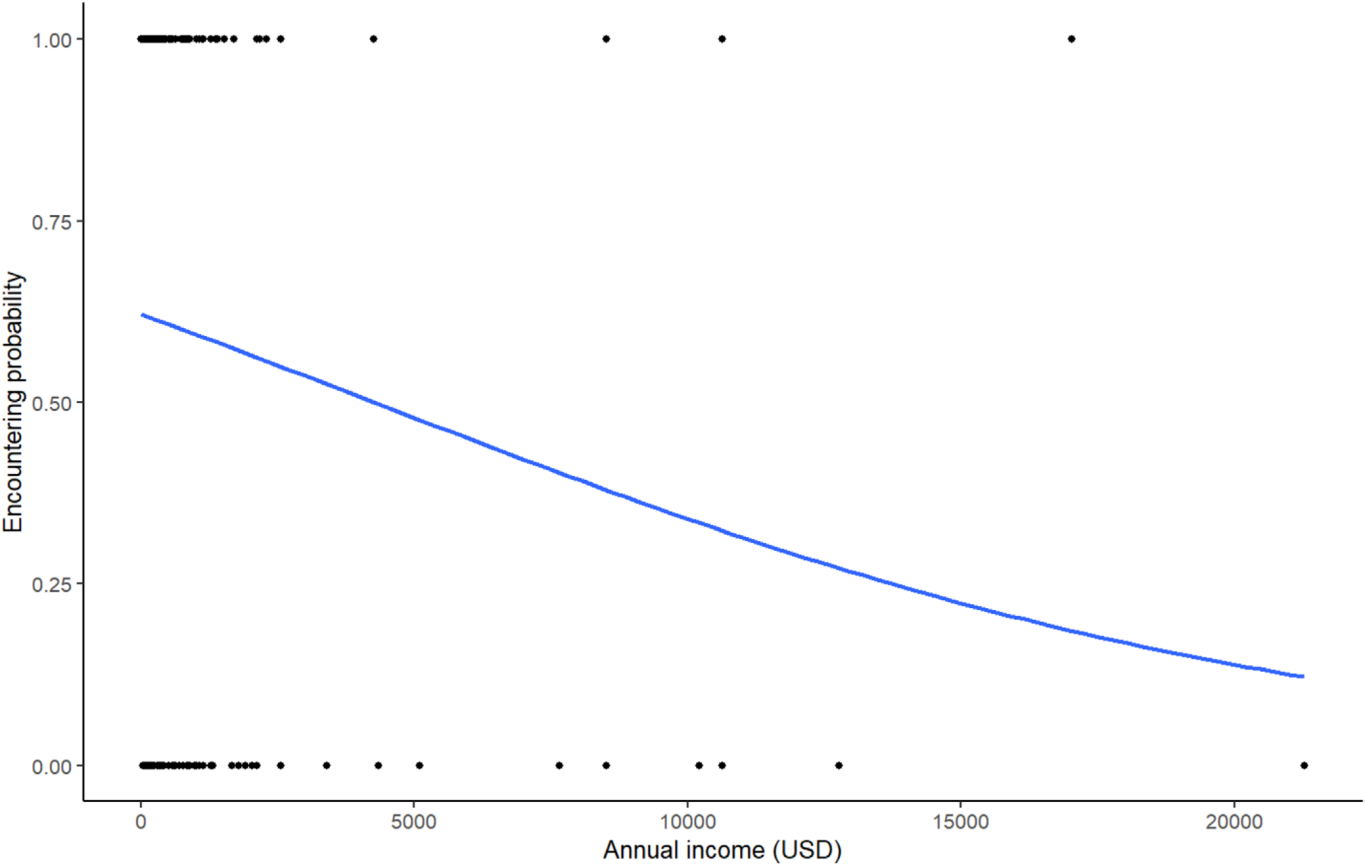
Relationships between the probability of encountering pangolins with an annual household income. The currency unit is the United States dollar.

Further, none of the human demographic factors were strongly related to pangolin observations (Table 1). Annual income showed a marginal effect on pangolin observations, with pangolin observations decreasing as income increased (GLM, binomial, *Z* = −1.78, *p* = 0.08, Table 1, Figure 3).

**TABLE 1:** Regression results for the effects of demographic variables on encountering pangolins.

Variable	Estimate	Standard error	*z*‐Value	*p*
(Intercept)	−0.515	0.821	−0.63	0.53
Gender: Male	0.312	0.437	0.71	0.48
Age	0.008	0.009	0.91	0.36
Occupation: Business	14.718	623.656	0.02	0.98
Occupation: Crop farmer	0.243	0.533	0.46	0.65
Occupation: Herbalist	14.202	882.744	0.02	0.99
Occupation: Pastoralist	0.067	0.571	0.12	0.91
Income	−0.042	0.024	−1.78	0.08
Residence years	−0.003	0.007	−0.42	0.68
Education	0.264	0.272	0.97	0.33

*Note:* Gender, Occupation, and Education are treated as factors; the “intercept” condition is Gender = Female, and Occupation = Agropastoral.

### 3.3 Group Size, Activity Pattern, and Observation Trends

The mean group size of pangolins observed by respondents is 0.91 ± 0.54. Regression results suggest that the mean reported group size did not differ between different categories of land use; however, among human activities, Patrol and Poaching resulted in a moderate negative effect on group size (GLM, quasi‐Poisson, *p* = 0.03; Table 2).

**TABLE 2:** Effects of human activity and land management type on the group size of pangolins reportedly observed.

Variable	Estimate	Standard error	*t*‐Value	*p*
(Intercept)	0.065	0.114	0.58	0.57
Land management: VL	−0.154	0.121	−1.27	0.21
Land management: WMA	0.154	0.147	1.05	0.30
Activity category: Medicine	−0.691	0.407	−1.7	0.09
Activity category: Opportunistic	0.068	0.092	0.74	0.46
Activity category: Patrol and poaching	−0.393	0.180	−2.18	0.03

*Note:* Intercept condition: Activity = Livelihood and Area = National Park.

**FIGURE 4:**
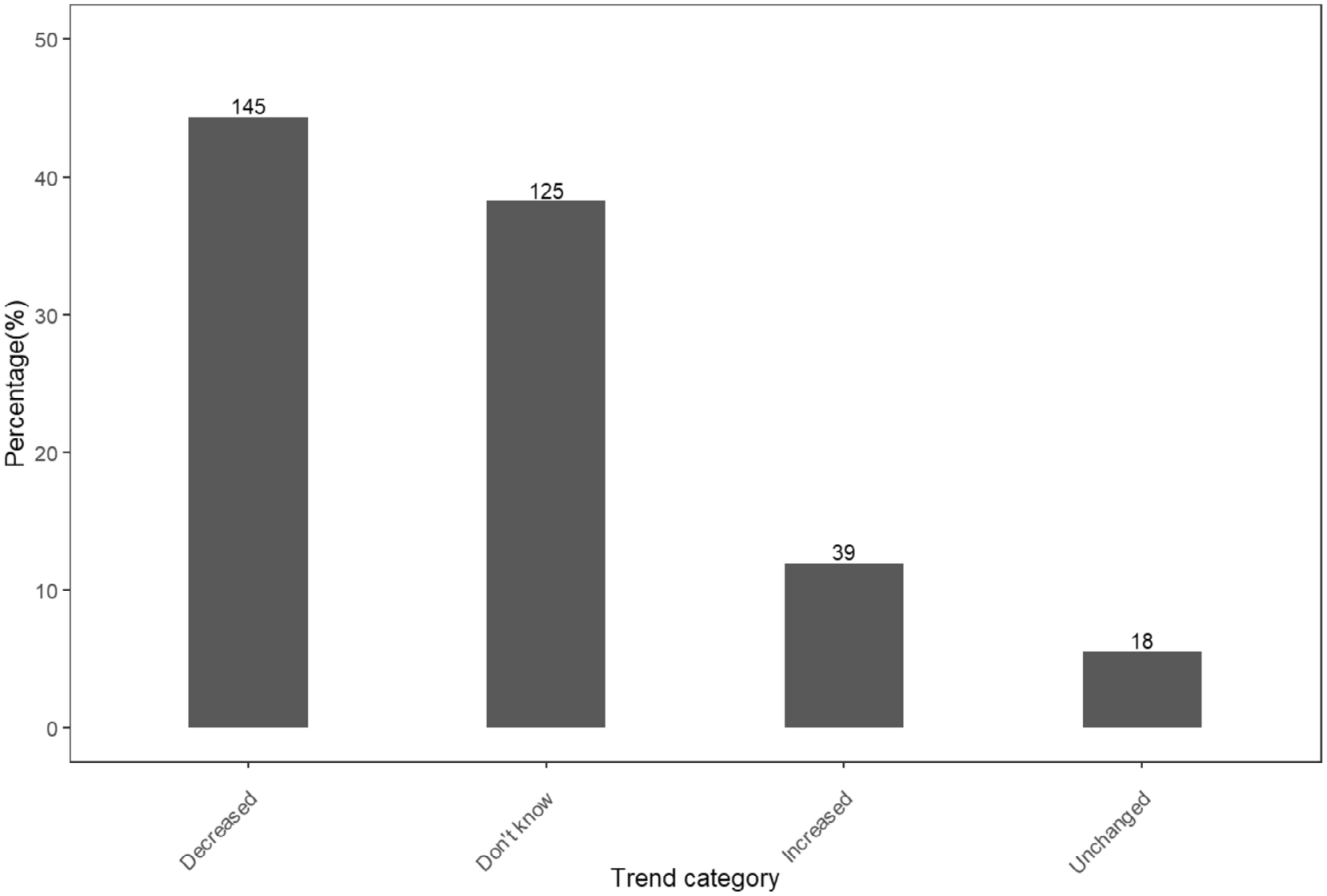
Reported trend in the frequency of pangolin encounters according to respondents during the survey conducted in villages found adjacent to Ruaha National Park. The numbers above the bars represent the number of responses for the given category.

We also assessed the trend in the frequency of pangolin observations over the past 5 years and found the response varied significantly (*χ* ^2^ = 42.4, df = 3, *p* < 0.001). Approximately 44% of respondents (*n* = 327) reported that the frequency of observing pangolins had decreased, whereas 38% were unaware of any trend in pangolin observations (Figure 4).

**FIGURE 5:**
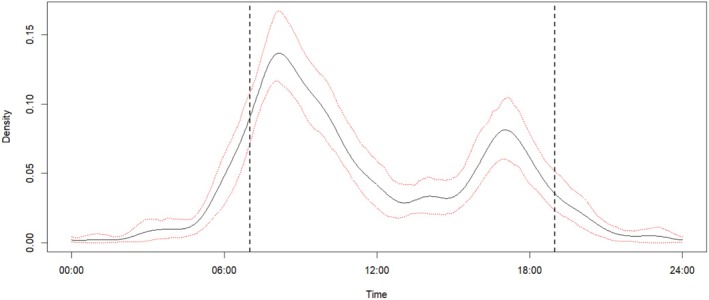
Daily circular distribution of hours that local people encountered pangolins. Black lines indicate the observed proportion of time (hours) of reported encounters, and the dotted red lines indicate the 95% confidence intervals. Vertical dotted black lines indicate sunrise and sunset times in the Ruaha landscape.

The daily timing of pangolin observations varied across the 24‐h clock, with most observations occurring in the morning (7–8 am) and evening (4–5 pm) hours (Figure 5).

## 4 Discussion

Working with local communities to provide conservation researchers with information critical to understanding wildlife populations and habitat needs is a cornerstone of practical conservation science (Dawson et al. 2021; Hughes, Elmeligi, and Morehouse 2022; Hughes, Tremblett, et al. 2022). As found with other studies in Tanzania, Cameroon, and Nepal, local community members can provide much‐needed information on pangolins, which can be used to enhance planning, monitoring, and applied conservation efforts (Fopa et al. 2020; Suwal et al. 2022; Walsh 2007). This is particularly true in areas with insufficient data to develop and implement effective conservation strategies (Simo, Difouo, et al. 2024; Simo, Massoh, et al. 2024).

Specific to our study, we found that the large representation of the Hehe tribe in our research was partly due to historically living in this area before Tanzania's resettlement processes (Kimaro and Hughes 2023; Walsh 2007). We also learned that pangolin sightings were expectedly related to where and when humans spent time, crop farming, and livestock grazing (Mrosso et al. 2022a, 2022b; Swiacká et al. 2022). We also found that men in our study, specifically those from lower‐income households, encountered pangolins more often, which could be related to seeking out natural resources to harvest (e.g., firewood, illegal wildlife hunting; Mrosso et al. 2022a, 2022b; Mgeni, Kicheleri, et al. 2024). However, we did not expect the mostly nocturnal pangolin to be observed during the daytime, as reported by our study participants.

As is consistent with other studies, the traditional livelihood activities of people in our research, combined with the limited options for generating income, result in local reliance on natural resources found in the village land or adjacent to protected areas (Mgemi et al. 2024; Walsh 2007). This means people spend the majority of their time engaging in crop cultivation, tending grazing livestock, collecting honey, gathering firewood, and possibly hunting wildlife—activities that are also associated with pangolin observations (Mrosso et al. 2022a, 2022b). However, we found that as income increased, the time spent harvesting natural resources decreased, and unsurprisingly, so did pangolin observations. We also noted that the frequency of pangolin observations decreased over the past 5 years, suggesting there may be a negative impact from harvesting natural resources on pangolins, including the potential for hunting and illegal trafficking, as well as habitat loss (Bhandari et al. 2025; Mgeni, Kicheleri, et al. 2024; Emogor et al. 2025).

As a starting point to address these challenges, we suggest engaging local communities in capacity‐building programs that have the potential to build on existing local knowledge and appreciation of pangolins, but also help support the species' conservation and help address human livelihood needs (Hughes et al. 2018). This can include training in scientific monitoring methods, whereby programs are designed to weave local people's traditional knowledge together with western science, to enable the collection of reliable and robust data on pangolins (Cooney et al. 2017; Hughes, Elmeligi, and Morehouse 2022; Hughes, Tremblett, et al. 2022; Roe et al. 2015). A local Tanzanian example of long‐term community engagement in monitoring wildlife includes the Lion Landscapes Community Camera Trapping Plus (CCT+) initiative in Ruaha (Dickman et al. 2025). This program has worked with local communities to monitor wildlife on their village lands using camera traps, and in return they receive points for rare or conflict‐prone species that are captured on camera; these points are then converted into tangible healthcare, education, or infrastructure benefits, thereby directly linking the conservation of wildlife species across village lands to peoples' well‐being (Dickman et al. 2025). The program has even evolved a step further, whereby CCT+ now rewards proactive conservation behaviors, including fortifying cattle enclosures to protect against depredation, and penalizes harmful actions, such as poisoning carcasses or snaring wildlife (Dickman et al. 2025). Overall, the lessons learned from CCT+ could be helpful in a pangolin conservation context, as demonstrated by other studies (Difouo, Simo, Kekeunou, Olson, and Ingram 2023; Simo, Difouo, Kekeunou, Olson, et al. 2023), where community agreements with local NGOs for monitoring and stewardship initiatives can help ensure direct benefits in a socially and ecologically relevant way.

Another pressing need for pangolins is ensuring they have a viable habitat that is maintained long‐term (Shirley et al. 2023). As such, we also suggest that training in community‐led land use planning can support both human agricultural production needs alongside habitat provisioning for pangolins, particularly in settings with dense and increasing human populations (Kiria et al. 2014). For example, the United States Agency for International Development's (USAID) program “*Partnership with the East African Community*” focused on building skills among local communities in land use planning and sustainability, to enhance natural resource management (USAID 2016). Likewise, the Food and Agriculture Organization (FAO) contributed efforts to planning for sustainable management, to address the challenges of human population growth, land degradation, and biodiversity loss (Ziadat et al. 2017).

Building capacity in community‐led land use planning has documented benefits, including transforming local development into collaborative efforts and moving away from top‐down (i.e., government‐led) decision‐making (Reed 2008; Kiria et al. 2014; Metternicht 2018). Advantages of engaging local communities in their own land use planning efforts can include weaving together local people's place‐based knowledge with western scientific methods to help enhance existing practices and contribute to conservation initiatives; this can consist of addressing illegal pangolin hunting (Simo, Difouo, Kekeunou, Ichu, et al. 2023), habitat degradation or loss (Reed 2008; Roe et al. 2015; Simo, Difouo, et al. 2024), as well as understanding feeding behavior and prey selection (Difouo, Simo, Kekeunou, Ebangue, et al. 2023; Difouo et al. 2024). Community‐led land use planning can foster a sense of ownership and incorporate diverse voices and needs, including those of women and youth (Chigbu 2020). Lastly, community‐led land use planning can build social capital, including trust, respect, and reciprocity through joint co‐learning initiatives between local people sitting together with government officials and non‐government organizations (Aditya et al. 2021; Hughes et al. 2023; Pretty and Smith 2004; Stern and Humphries 2022). Indeed, as suggested by Shirley et al. (2023), conservation action must incorporate community‐based monitoring efforts together with addressing illegal hunting and trafficking, and notably, habitat restoration.

We suggest that if these opportunities were to exist, it is possible that threats to pangolins from illegal harvesting could decrease, given that local people could transition from a heavy reliance on traditional subsistence livelihoods (which increase human‐pangolin encounter rates) to alternative employment and income‐generating opportunities (i.e., conservation jobs, sustainable farming practices, etc.; Brashares et al. 2011; Roe et al. 2015). That said, understanding the benefits of community engagement in monitoring or land use planning, among other sustainable alternative livelihood programs specific to pangolin conservation is lacking (Abu‐Bakarr et al. 2022; O'Connell et al. 2022; Roe et al. 2015). Future work must therefore develop evaluative techniques and relevant indicators to assess their effectiveness for both people and pangolins.

## 5 Conclusions

As conservation scientists, we are cognizant of our desire to seek the protection and maintenance of pangolins in Tanzania and beyond. At the same time, we also aim to support the livelihoods and well‐being of the people we work with in conservation. As such, conservation solutions must account for the variety of values, needs, and desired outcomes that local people have for species like pangolins. To us, this means ensuring that local communities are part of conservation decision‐making and action‐oriented processes, working alongside government institutions and conservation organizations, to design appropriate management options that help ensure sustainable human land use and long‐term pangolin populations in Tanzania. By working together, we can co‐create knowledge and skills related to pangolin conservation and build meaningful cooperative relationships across local communities, conservationists, and managers (Sibanda et al. 2021). We hope that this study contributes to meaningful, applied conservation action for pangolins in Tanzania, with explicit acknowledgment and outcomes for the people who live alongside them.

## Author Contributions

**Rose Peter Kicheleri:** conceptualization (equal), funding acquisition (equal), investigation (equal), methodology (equal). **Courtney Hughes:** conceptualization (equal), methodology (equal), validation (equal), writing – review and editing (equal). **Michael Honorati Kimaro:** conceptualization (equal), data curation (equal), formal analysis (equal), funding acquisition (equal), methodology (equal), project administration (equal), visualization (equal), writing – original draft (equal), writing – review and editing (equal). **Charles Peter Mgeni:** conceptualization (equal), data curation (equal), funding acquisition (equal), investigation (equal), project administration (equal). **Nyemo Amos Chilagane:** conceptualization (equal), methodology (equal), project administration (equal), writing – original draft (equal). **Hillary Thomas Mrosso:** conceptualization (equal), data curation (equal), investigation (equal), methodology (equal), resources (equal), writing – original draft (equal). **Simon Joshua Chidodo:** conceptualization (equal), methodology (equal), writing – review and editing (equal). **Fenrick Filbert Msigwa:** conceptualization (equal), data curation (equal), investigation (equal), resources (equal), writing – review and editing (equal). **Elisante Azaeli Kimambo:** conceptualization (equal), writing – review and editing (equal). **Rajabu Joseph Kangile:** conceptualization (equal), data curation (equal), formal analysis (equal), methodology (equal), writing – review and editing (equal). **George Bunyata Bulenga:** conceptualization (equal), data curation (equal), writing – review and editing (equal). **Camille Warbington:** conceptualization (equal), formal analysis (equal), writing – original draft (equal).

## Ethics Statement

The university's ethical committee approved the survey questionnaire. The study complied with the Sokoine University of Agriculture in Tanzania's 2019 research standards and guidelines.

## Conflicts of Interest

The authors declare no conflicts of interest.

## Supporting information


**Appendix S1:** ece371987‐sup‐0001‐AppendixS1.zip.

## Data Availability

Data and R codes used in this manuscript have been shared and can be accessed as supporting material via the following link: https://drive.google.com/drive/folders/11sEl44QlPAonDX8lN7dZYVJDnn6NlnIS?usp=drive_link.

## References

[ece371987-bib-0069] Abade, L. , D. W. Macdonald , and A. J. Dickman . 2014. “Using Landscape and Bioclimatic Features to Predict the Distribution of Lions, Leopards and Spotted Hyaenas in Tanzania's Ruaha Landscape.” PLoS One 9, no. 5: e96261. 10.1371/journal.pone.0096261. 24797792 PMC4010451

[ece371987-bib-0001] Abu‐Bakarr, I. , M. I. Bakarr , N. Gelman , et al. 2022. “Capacity and Leadership Development for Wildlife Conservation in Sub‐Saharan Africa: Assessment of a Programme Linking Training and Mentorship.” Oryx 56, no. 5: 744–752. 10.1017/S0030605321000855.

[ece371987-bib-0002] Aditya, V. , K. P. Komanduri , R. Subhedar , and T. Ganesh . 2021. “Integrating Camera Traps and Community Knowledge to Assess the Status of the Indian Pangolin *Manis crassicaudata* in the Eastern Ghats, India.” Oryx 55, no. 5: 677–683. 10.1017/S0030605319001303.

[ece371987-bib-0003] Baiyewu, A. O. , M. K. Boakye , A. Kotzé , D. L. Dalton , and R. Jansen . 2018. “Ethnozoological Survey of Traditional Uses of Temminck's Ground Pangolin (*Smutsia temminckii*) in South Africa.” Society and Animals 26, no. 3: 306–307. 10.1163/15685306-12341515.

[ece371987-bib-0004] Bergen, N. , and R. Labonté . 2020. ““Everything Is Perfect, and We Have No Problems”: Detecting and Limiting Social Desirability Bias in Qualitative Research.” Qualitative Health Research 30, no. 5: 783–792. 10.1177/1049732319889354. 31830860

[ece371987-bib-0005] Bhandari, B. , B. Dhami , N. KC , et al. 2025. “Habitat and Anthropogenic Determinants of Chinese Pangolin ( *Manis pentadactyla* ) Burrow Occupancy in Udayapur, Eastern Nepal: Implications for Site‐Specific Conservation.” Ecology and Evolution 15, no. 6: e71493. 10.1002/ece3.71493. 40469467 PMC12134491

[ece371987-bib-0007] Brashares, J. S. , C. D. Golden , K. Z. Weinbaum , C. B. Barrett , and G. V. Okello . 2011. “Economic and Geographic Drivers of Wildlife Consumption in Rural Africa.” Proceedings of the National Academy of Sciences of the United States of America 108, no. 34: 13931–13936. 10.1073/pnas.1011526108. 21873180 PMC3161600

[ece371987-bib-0008] Brittain, S. , M. Ngo Bata , P. De Ornellas , E. J. Milner‐Gulland , and M. Rowcliffe . 2020. “Combining Local Knowledge and Occupancy Analysis for a Rapid Assessment of the Forest Elephant *Loxodonta cyclotis* in Cameroon's Timber Production Forests.” Oryx 54, no. 1: 90–100. 10.1017/S0030605317001569.

[ece371987-bib-0010] Camino, M. , J. Thompson , L. Andrade , S. Cortez , S. D. Matteucci , and M. Altrichter . 2020. “Using Local Ecological Knowledge to Improve Large Terrestrial Mammal Surveys, Build Local Capacity and Increase Conservation Opportunities.” Biological Conservation 244: 108450. 10.1016/j.biocon.2020.108450.

[ece371987-bib-0011] Chigbu, U. E. 2020. “Land, Women, Youths, and Land Tools or Methods: Emerging Lessons for Governance and Policy.” Land 9, no. 12: 507. 10.3390/land9120507.

[ece371987-bib-0012] Cooney, R. , D. Roe , H. Dublin , et al. 2017. “From Poachers to Protectors: Engaging Local Communities in Solutions to Illegal Wildlife Trade.” Conservation Letters 10, no. 3: 367–374. 10.1111/conl.12294.

[ece371987-bib-0071] Creswell, J. W. , and J. D. Creswell . 2023. Research Design: Qualitative, Quantitative, and Mixed Methods Approaches. 6th ed. SAGE Publications, Inc.

[ece371987-bib-0013] Cusack, J. J. , A. J. Dickman , J. M. Rowcliffe , C. Carbone , D. W. Macdonald , and T. Coulson . 2015. “Random Versus Game Trail‐Based Camera Trap Placement Strategy for Monitoring Terrestrial Mammal Communities.” PLoS One 10, no. 5: e0126373. 10.1371/journal.pone.0126373. 25950183 PMC4423779

[ece371987-bib-0014] Dawson, M. N. , B. Coolsaet , E. J. Sterling , et al. 2021. “The Role of Indigenous Peoples and Local Communities in Effective and Equitable Conservation.” Ecology and Society 26, no. 3: 19. 10.5751/ES-12625-260319.

[ece371987-bib-0015] D'Cruze, N. , B. Singh , A. Mookerjee , L. A. Harrington , and D. W. Macdonald . 2018. “A Socio‐Economic Survey of Pangolin Hunting in Assam, Northeast India.” Nature Conservation 30: 83–105. 10.3897/natureconservation.30.27379.

[ece371987-bib-0016] Dickman, A. , A. Cotterill , S. Asecheka , et al. 2025. “Community Camera Trapping: A Novel Method for Encouraging Human‐Big Cat Coexistence on Human‐Dominated Land.” Wildlife Letters 3, no. 1: 22–29. 10.1002/wll2.70006.

[ece371987-bib-0017] Difouo, G. F. , F. T. Simo , S. Kekeunou , et al. 2023. “Diversity Patterns of Ants and Termites in Forest‐Savanna Mosaic Habitats in Two Protected Areas of Cameroon.” African Journal of Ecology 61, no. 4: 840–859. 10.1111/aje.13183.

[ece371987-bib-0018] Difouo, G. F. , F. T. Simo , S. Kekeunou , O. R. Fokou , L. G. Ndoh , and D. Olson . 2024. “Ants (Hymenoptera: Formicidae) and Termites (Blattodea: Termitoidae) in the Diet of Wild White‐Bellied Pangolin (*Phataginus tricuspis*) in Forest‐Savanna Habitats of Cameroon.” Zoo Biology 43, no. 4: 315–324. 10.1002/zoo.21834. 38685797

[ece371987-bib-0019] Difouo, G. F. , F. T. Simo , S. Kekeunou , D. Olson , and D. J. Ingram . 2023. “Black‐Bellied Pangolin *Phataginus tetradactyla* Documented in Deng Deng National Park, Cameroon, Using Camera Traps.” Oryx 57, no. 6: 701–703. 10.1017/S0030605323000352.

[ece371987-bib-0020] Emogor, C. A. , S. K. Wasser , L. Coad , et al. 2025. “Pangolin Hunting in Southeast Nigeria Is Motivated More by Local Meat Consumption Than International Demand for Scales.” Nature Ecology & Evolution 9: 1349–1358. 10.1038/s41559-025-02734-3. 40514569 PMC12328206

[ece371987-bib-0021] Foley, C. , D. De Luca , T. R. B. Davenport , et al. 2014. A Field Guide to the Larger Mammals of Tanzania. Princeton University Press. http://site.ebrary.com/id/10872420.

[ece371987-bib-0022] Fopa, G. D. , F. Simo , S. Kekeunou , I. G. Ichu , D. J. Ingram , and D. Olson . 2020. “Understanding Local Ecological Knowledge, Ethnozoology, and Public Opinion to Improve Pangolin Conservation in the Center and East Regions of Cameroon.” Journal of Ethnobiology 40, no. 2: 234–251. 10.2993/0278-0771-40.2.234.

[ece371987-bib-0023] George, T. , and R. Y. M. Kangalawe . 2024. “The Impact of Climate Change on Livelihoods of Communities Adjacent to Protected Areas in the Ruaha‐Rungwa Landscape.” Journal of the Geographical Association of Tanzania 44, no. 1: 85–107. 10.56279/jgat.v44i1.284.

[ece371987-bib-0024] Hallwass, G. , A. Schiavetti , and R. A. M. Silvano . 2020. “Fishers' Knowledge Indicates Temporal Changes in Composition and Abundance of Fishing Resources in Amazon Protected Areas.” Animal Conservation 23, no. 1: 36–47. 10.1111/acv.12504.

[ece371987-bib-0025] Hariohay, M. K. , et al. 2023. “Ethnozoological Uses of Wild Animals Among the Iraqw in Northern Tanzania.” Tropical Zoology 36, no. 1–2: 36–52. 10.4081/tz.2023.131.

[ece371987-bib-0026] Heighton, S. P. , and P. Gaubert . 2021. “A Timely Systematic Review on Pangolin Research, Commercialization, and Popularization to Identify Knowledge Gaps and Produce Conservation Guidelines.” Biological Conservation 256: 109042. 10.1016/j.biocon.2021.109042.

[ece371987-bib-0072] Hughes, A. , M. Auliya , S. Altherr , et al. 2023. “Determining the Sustainability of Legal Wildlife Trade.” Journal of Environmental Management 341: 117987. 10.1016/j.jenvman.2023.117987. 37178541

[ece371987-bib-0027] Hughes, C. , S. Elmeligi , and A. Morehouse . 2022. “Conservation Through Connection: Approaches to Engaging Communities in Applied Grizzly Bear Research.” Frontiers in Conservation Science 3: 913668. 10.3389/fcosc.2022.913668.

[ece371987-bib-0030] Hughes, C. , L. Marker , K. Marnewick , L. K. Boast , and A. Schmidt‐Küntzel . 2018. “Chapter 18 – Cheetah Conservation and Educational Programs.” In Cheetahs: Biology and Conservation, edited by P. R. Krausman and S. M. Morales , 249–259. Academic Press. 10.1016/B978-0-12-804088-1.00018-6.

[ece371987-bib-0028] Hughes, C. , and S. E. Nielsen . 2019. “‘Bear Are Only the Lightning Rod’: Ongoing Acrimony in Alberta's Grizzly Bear Recovery.” Society & Natural Resources 32, no. 1: 34–52. 10.1080/08941920.2018.1502853.

[ece371987-bib-0029] Hughes, C. , K. Tremblett , J. Kummer , T. S. Lee , and D. Duke . 2022. “How Can We Do Citizen Science Better? A Case Study Evaluating Grizzly Bear Citizen Science Using Principles of Good Practice in Alberta, Canada.” Animals 12, no. 9: 1068. 10.3390/ani12091068. 35565495 PMC9102148

[ece371987-bib-0031] Hughes, C. , N. Yarmey , A. Morehouse , and S. Nielsen . 2020. “Problem Perspectives and Grizzly Bears: A Case Study of Alberta's Grizzly Bear Recovery Policy.” Frontiers in Ecology and Evolution 8: 38. 10.3389/fevo.2020.00038.

[ece371987-bib-0032] Katuwal, H. B. , H. P. Sharma , and K. Parajuli . 2017. “Anthropogenic Impacts on the Occurrence of the Critically Endangered Chinese Pangolin ( *Manis pentadactyla* ) in Nepal.” Journal of Mammalogy 98, no. 6: 1667–1673. 10.1093/jmammal/gyx114.

[ece371987-bib-0033] Kimaro, M. H. , and C. Hughes . 2023. “Conditions of Conflict: Exploring Pastoralist Resettlement in Relation to African Lion Conservation.” Society & Natural Resources 38, no. 4: 352–373. 10.1080/08941920.2023.2263861.".

[ece371987-bib-0034] Kiria, E. M. , J. N. Ayonga , and H. Ipara . 2014. “Promoting Effective Community Participation in Land Use Planning and Management of Wildlife Conservation Areas.” Journal of Natural Science Research 4, no. 20: 1–10.

[ece371987-bib-0074] Lavrakas, P. J. , ed. 2008. Encyclopedia of Survey Research Methods. SAGE Publications. http://www.credoreference.com/book/sagesurveyr.

[ece371987-bib-0036] Metternicht, G. 2018. Land Use and Spatial Planning: Enabling Sustainable Management of Land Resources. Springer. 10.1007/978-3-319-71861-3.

[ece371987-bib-0075] Mgeni, C. P. , C. Kiffner , T. Caro , et al. 2024. “Illegal Harvest, Use, and Trade in Temminck's Pangolins by Communities Adjacent to Ruaha National Park, Tanzania.” Human Dimensions of Wildlife 29, no. 5: 1–14. 10.1080/10871209.2024.2435298.

[ece371987-bib-0076] Microsoft Corporation . 2021. Microsoft Office Professional Plus 2021 [Computer Program]. Microsoft Corporation.

[ece371987-bib-0038] Mrosso, H. T. , R. P. Kicheleri , J. J. Kashaigili , et al. 2022a. “Illegal Wildlife Trade: Trade Flows of Wildlife Products and Facilitation Methods in the Ruaha Landscape, Tanzania.” Open Journal of Ecology 12, no. 09: 585–603. 10.4236/oje.2022.129033.

[ece371987-bib-0039] Mrosso, H. T. , R. P. Kicheleri , J. J. Kashaigili , et al. 2022b. “Wildlife Poaching Practices in Tanzania's Ruaha Landscape.” Journal of Forestry and Nature Conservation 91, no. 2: 106–119.

[ece371987-bib-0040] Nixon, S. , D. Pietersen , D. Challender , et al. 2019. “*Smutsia gigantea*”. 10.2305/IUCN.UK.2019-3.RLTS.T12762A123584478.en.

[ece371987-bib-0041] O'Connell, M. , A. Donnison , K. Lynch , and R. Bennett . 2022. “Assessing National‐Level Provision of Conservation Capacity Building: Lessons Learnt From a Case Study of Kenya.” Oryx 56, no. 5: 753–759. 10.1017/S0030605322000345.

[ece371987-bib-0042] Patton, M. Q. 2002. Qualitative Research and Evaluation Methods. 3rd ed. Sage Publications.

[ece371987-bib-0043] Penrod, J. , D. B. Preston , R. E. Cain , and M. T. Starks . 2003. “A Discussion of Chain Referral as a Method of Sampling Hard‐to‐Reach Populations.” Journal of Transcultural Nursing: Official Journal of the Transcultural Nursing Society 14, no. 2: 100–107. 12772618 10.1177/1043659602250614

[ece371987-bib-0044] Perera, P. , and H. Karawita . 2020. “An Update of Distribution, Habitats and Conservation Status of the Indian Pangolin (*Manis crassicaudata*) in Sri Lanka.” Global Ecology and Conservation 21: e00799. 10.1016/j.gecco.2019.e00799. PMC6939095 31909106

[ece371987-bib-0045] Pietersen, D. , R. Jansen , and E. Connelly . 2019. “*Smutsia temminckii* .” 10.2305/IUCN.UK.2019-3.RLTS.T12765A123585768.en.

[ece371987-bib-0046] Pietersen, D. , et al. 2019. “*Phataginus tricuspis* .” 10.2305/IUCN.UK.2019-3.RLTS.T12767A123586469.en.

[ece371987-bib-0047] Potgieter, G. C. , F. J. Weise , B. Wachter , J. Melzheimer , I. Wiesel , and K. Stratford . 2017. “Comment on Rust Et al.: Human–Carnivore Conflict in Namibia Is Not Simply About Black and White.” Society & Natural Resources 30, no. 10: 1299–1303. 10.1080/08941920.2017.1283077.

[ece371987-bib-0048] Pretty, J. , and D. Smith . 2004. “Social Capital in Biodiversity Conservation and Management.” Conservation Biology 18, no. 3: 631–638. 10.1111/j.1523-1739.2004.00126.x.

[ece371987-bib-0049] R Core Team . 2021. R: A Language and Environment for Statistical Computing. R Foundation for Statistical Computing. https://www.R‐project.org/.

[ece371987-bib-0050] Reed, M. S. 2008. “Stakeholder Participation for Environmental Management: A Literature Review.” Biological Conservation 141, no. 10: 2417–2431. 10.1016/j.biocon.2008.07.014.

[ece371987-bib-0051] Roe, D. , F. Booker , M. Day , et al. 2015. “Are Alternative Livelihood Projects Effective at Reducing Local Threats to Specified Elements of Biodiversity and/or Improving or Maintaining the Conservation Status of Those Elements?” Environmental Evidence 4, no. 1: 22. 10.1186/s13750-015-0048-1.

[ece371987-bib-0052] Rust, N. A. , A. Abrams , D. W. S. Challender , et al. 2017. “Quantity Does Not Always Mean Quality: The Importance of Qualitative Social Science in Conservation Research.” Society & Natural Resources 30, no. 10: 1304–1310. 10.1080/08941920.2017.1333661.

[ece371987-bib-0053] Shirley, M. H. , M. H. Shirley , G. Gerard , E. Panjang , N. C.‐M. Sun , and S. P. Heighton . 2023. “Pangolins: Epitomizing the Complexities of Conservation.” Oryx 57, no. 6: 681–682. 10.1017/S0030605323001655.

[ece371987-bib-0054] Sibanda, L. , E. van der Meer , P. J. Johnson , et al. 2021. “Evaluating the Effects of a Conservation Intervention on Rural Farmers' Attitudes Toward Lions.” Human Dimensions of Wildlife 26, no. 5: 445–460. 10.1080/10871209.2020.1850933.

[ece371987-bib-0058] Simo, F. T. , L. Coad , J. K. Nyumu , G. Mbeh , and E. E. Abwe . 2024. “Local Knowledge, Uses, and Conservation Status of the African Golden Cat Caracal Aurata in Central Cameroon.” Human Dimensions of Wildlife 29, no. 5: 1–16. 10.1080/10871209.2024.2389998.

[ece371987-bib-0055] Simo, F. T. , G. F. Difouo , S. Kekeunou , I. G. Ichu , D. J. Ingram , and D. Olson . 2023. “Pangolin Hunting and Trafficking in the Forest‐Savannah Transition Area of Cameroon.” Oryx 57, no. 6: 704–713. 10.1017/S0030605322001429.

[ece371987-bib-0056] Simo, F. T. , G. F. Difouo , S. Kekeunou , et al. 2023. “Adapting Camera‐Trap Placement Based on Animal Behavior for Rapid Detection: A Focus on the Endangered, White‐Bellied Pangolin (*Phataginus tricuspis*).” Ecology and Evolution 13, no. 5: e10064. 10.1002/ece3.10064. PMC10172612 37181204

[ece371987-bib-0057] Simo, F. T. , G. F. Difouo , C. N. Tchana , et al. 2024. “Urban Wild Meat and Pangolin Consumption Across Southern Forested Cameroon: The Limited Influence of COVID‐19.” *People and Nature* [Preprint]. 10.1002/pan3.10634.

[ece371987-bib-0059] Sokoine University of Agriculture (SUA) . 2019. Research Regulations and Guidelines. https://www.dprtc.sua.ac.tz/wp‐content/uploads/SUA‐Research‐Regulations‐and‐Guidelines‐2019.pdf.

[ece371987-bib-0060] Stern, E. R. , and M. M. Humphries . 2022. “Interweaving Local, Expert, and Indigenous Knowledge Into Quantitative Wildlife Analyses: A Systematic Review.” Biological Conservation 266: 109444. 10.1016/j.biocon.2021.109444.

[ece371987-bib-0061] Suwal, T. L. , S. Gurung , M. Bakhunchhe Shrestha , D. J. Ingram , and K. J. C. Pei . 2022. “Human Dimensions of Pangolin Conservation: Indigenous and Local Knowledge, Ethnozoological Uses, and Willingness of Rural Communities to Enhance Pangolin Conservation in Nepal.” Journal of Ethnobiology 42, no. 3: 1–18. 10.2993/0278-0771-42.3.7.

[ece371987-bib-0062] Swiacká, M. , D. J. Ingram , T. Bohm , and F. Ceacero . 2022. “Perceptions and Uses of Pangolins (Pholidota) Among Remote Rural Communities in the Republic of the Congo: A Baseline Study From the Odzala‐Kokoua National Park.” Conservation Science and Practice 4, no. 12: e12839. 10.1111/csp2.12839.

[ece371987-bib-0063] The United Republic of Tanzania (URT) . 2009. “The Wildlife Conservation Act No. 5.” https://www.oaklandinstitute.org/sites/oaklandinstitute.org/files/pdfpreview/wildlife_conservation_act_2009_tanzania_0.pdf.

[ece371987-bib-0064] Trageser, S. J. , A. Ghose , M. Faisal , et al. 2017. “Pangolin Distribution and Conservation Status in Bangladesh.” PLoS One 12, no. 4: e0175450. 10.1371/journal.pone.0175450. 28388644 PMC5384767

[ece371987-bib-0077] Traoré, S. , and M. Lepage . 2008. “Effects of Controlled Livestock Grazing and Annual Prescribed Fire on Epigeal Termite Mounds in a Savannah Woodland in Burkina Faso.” Insectes Sociaux 55, no. 2: 183–189. 10.1007/s00040-008-0998-1.

[ece371987-bib-0065] USAID . 2016. “USAID Partnership With the East African Community: Sustainability and Transition Planning for Regional Integration.” East African Community. https://abcg.org/wp‐content/uploads/2023/08/ABCG‐ICCB_LUM‐2023.pdf.

[ece371987-bib-0066] Walsh, M. T. 2007. “Pangolins and Politics in the Great Ruaha Valley, Tanzania: Symbol, Ritual, and Difference [Pangolin et politique dans la vallée du Great Ruaha, Tanzanie: symbole, rituel et différence].” In Animal Symbolism: Animals, Keystone of the Relationship Between Man and Nature? [Le Symbolisme Des Animaux L'animal, Clef de voûte de la Relation Entre L'homme et la Nature], edited by E. Dounias , E. Motte‐Florac , and M. Dunham , 1003–1044. Éditions de l'IRD. http://horizon.documentation.ird.fr/exl‐doc/pleins_textes/divers16‐08/010041952.pdf.

[ece371987-bib-0067] Willcox, D. , H. C. Nash , S. Trageser , et al. 2019. “Evaluating Methods for Detecting and Monitoring Pangolin (Pholidata: Manidae) Populations.” Global Ecology and Conservation 17: e00539. 10.1016/j.gecco.2019.e00539.

[ece371987-bib-0068] Ziadat, F. , S. Bunning , and E. De Pauw . 2017. Land Resource Planning for Sustainable Land Management: Current and Emerging Needs in Land Resource Planning for Food Security, Sustainable Livelihoods, Integrated Landscape Management and Restoration. Food and Agriculture Organization of the United Nations. https://openknowledge.fao.org/server/api/core/bitstreams/710fc58f‐00c6‐4855‐b4ea‐c455048bd49f/content.

