# Supplementary material for: Local Ecological Knowledge Insights Into the Distribution and Activity Patterns of Temminck's Pangolin in Ruaha Landscape, Tanzania
Source: Ecol Evol. 2025 Aug 16;15(8):e71987. doi: 10.1002/ece3.71987 (PMC12357171; doi:10.1002/ece3.71987)
Supplement: Supplementary file 1 — Appendix S1: ece371987‐sup‐0001‐AppendixS1.zip. [file ECE3-15-e71987-s001.zip › dataANDscript/LEK_Pangolin_Encounters_and_Activity_Clean_Code_Nov2024.pdf]

Data Analysis - Local ecological knowledge insights into the distribution and activity patterns of Temminck's grouns pangolin in Ruaha landscape, Tanzania

2024-11-16

```r
library(ggplot2)
library(readxl)
library(grid)
library(gridExtra)
library(chisq.posthoc.test)
library(ggcorrplot)
library(dplyr)
```

```
Attaching package: 'dplyr'

The following object is masked from 'package:gridExtra':

    combine

The following objects are masked from 'package:stats':

    filter, lag

The following objects are masked from 'package:base':

    intersect, setdiff, setequal, union
```

```r
library(ggpubr)
library(MASS)
```

```
Attaching package: 'MASS'


The following object is masked from 'package:dplyr':

    select
```

```r
library(plotrix)
library(stringr)
```

```r
#Clear R's brain
rm(list = ls())

#Where R is looking now
# getwd()

#Upload our dataset
mydata<-read_excel("Data2.xlsx", na = "NA")
str(mydata)
```

```
tibble [386 x 22] (S3: tbl_df/tbl/data.frame)
 $ Village          : chr [1:386] "Tungamalenga" "Tungamalenga" "Tungamalenga" "Tungamalenga"
 $ Tribe            : num [1:386] 2 5 8 8 8 8 5 8 5 5 ...
 $ Gender           : num [1:386] 1 1 1 1 1 1 1 1 1 1 ...
 $ Age              : num [1:386] 34 32 32 32 60 45 28 27 31 29 ...
 $ Occupation       : chr [1:386] "Agropastoralist" "Patoralist" "Patoralist" "Patoralist" ..
 $ Annual_Income    : num [1:386] 1500000 180000 4200000 4200000 200000 2000000 800000 100000
 $ Rounded_inc      : num [1:386] 1.5 0.18 4.2 4.2 0.2 2 0.8 1 2 0.4 ...
 $ Residence_years : num [1:386] 17 32 32 32 10 26 28 27 20 29 ...
 $ Education        : num [1:386] 2 2 3 3 1 1 3 2 2 3 ...
 $ Area_encountered: chr [1:386] "WMA" "VL" "PA" "PA" ...
 $ Habitat          : num [1:386] 1 3 5 5 3 3 4 3 3 3 ...
 $ Habitat_visit    : num [1:386] 26 52 52 52 52 52 52 52 1 52 ...
 $ Sighting_freq    : num [1:386] 1 1 1 1 1 1 1 1 1 1 ...
 $ Group_size       : num [1:386] 1 1 2 2 0 1 1 1 0 0 ...
 $ Hours            : POSIXct[1:386], format: "1899-12-31 08:00:00" "1899-12-31 17:00:00" ...
 $ Season           : num [1:386] 1 3 4 4 NA 4 4 4 NA NA ...
 $ Trend            : num [1:386] 3 2 2 2 NA 2 1 2 NA NA ...
 $ Vland            : num [1:386] 0 1 0 0 0 1 0 1 0 0 ...
 $ Area_VL          : num [1:386] NA 3 NA NA NA 3 NA 5 NA NA ...
 $ Activity         : chr [1:386] NA "Grazing" NA NA ...
 $ Landuse_effect   : num [1:386] 0 1 1 1 0 1 1 0 1 1 ...
 $ Reason           : num [1:386] 1 1 2 2 1 3 5 1 2 5 ...
```

Explore the types of areas where pangolins were encountered by the survey participants Options are: PA - Protected Area (the national park) WMA - Wildlife Management Area (managed by villagers) VL - Village Lands (where human settlement, crop cultivation and livestock grazing is allowed)

```
#Research Q1: counts vs land use types
#Create bar graph

landuse<-read_excel("Landuse.xlsx") ## count of Area_encountered in mydata
names(landuse)
```

```
[1] "Landuse"    "Counts"     "Percentage"
```

```
p1<-ggplot(landuse, aes(Landuse, Percentage)) +
  geom_bar(stat = "identity", width = 0.4) +
  theme_bw(base_size = 11) +
  theme(panel.grid.major = element_blank(),
        panel.grid.minor = element_blank()) +
  ylab("Percentage(%)") +
  xlab("Landuse category")+ggtitle("a")
p1
```

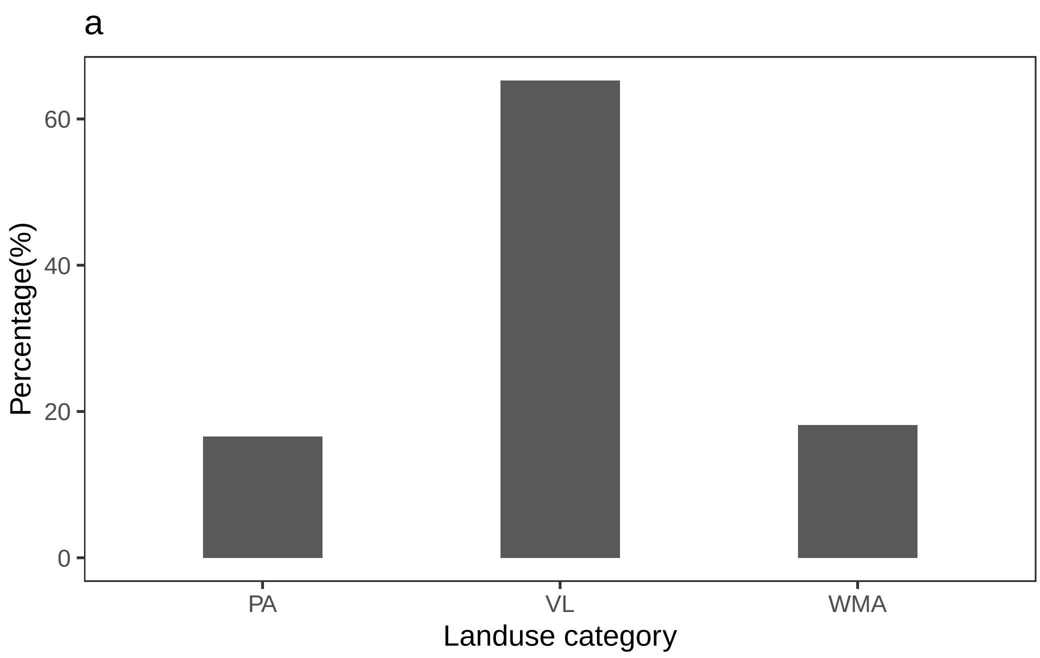


```
#Let's perform chi-square test
fcount<-matrix(c(17,18,65))
```

```r
f_chisq<-chisq.test(fcount)
f_chisq
```

```

    Chi-squared test for given probabilities

data:  fcount
X-squared = 45, df = 2, p-value = 2e-10
```

```r
## RESULTS: Significant differences found - X-squared = 45.14, df = 2, p-value = 1.578e-10
```

Next research question is to explore the types of activities that people were involved in when they encountered pangolins.

```r
## NOTE - THESE ARE THE UNCOMBINED ACTIVITY COUNTS
##Research Q2: counts vs land use types
#Create bar graph
activity<-read_excel("Activity.xlsx") ## column "Activity" in mydata
names(activity)
```

```
[1] "Activity"   "Count"      "Percentage"
```

```r
p2<-ggplot(activity, aes(Activity, Percentage)) +
  geom_bar(stat = "identity", width = 0.4)+theme_bw(base_size = 11) +
  theme(panel.grid.major = element_blank(),
        panel.grid.minor = element_blank()) +
  ylab("Percentage(%)")+xlab("")+ggtitle("b") +
  theme(axis.text.x = element_text(angle = 45, vjust = 0.8, hjust=1))
p2
```

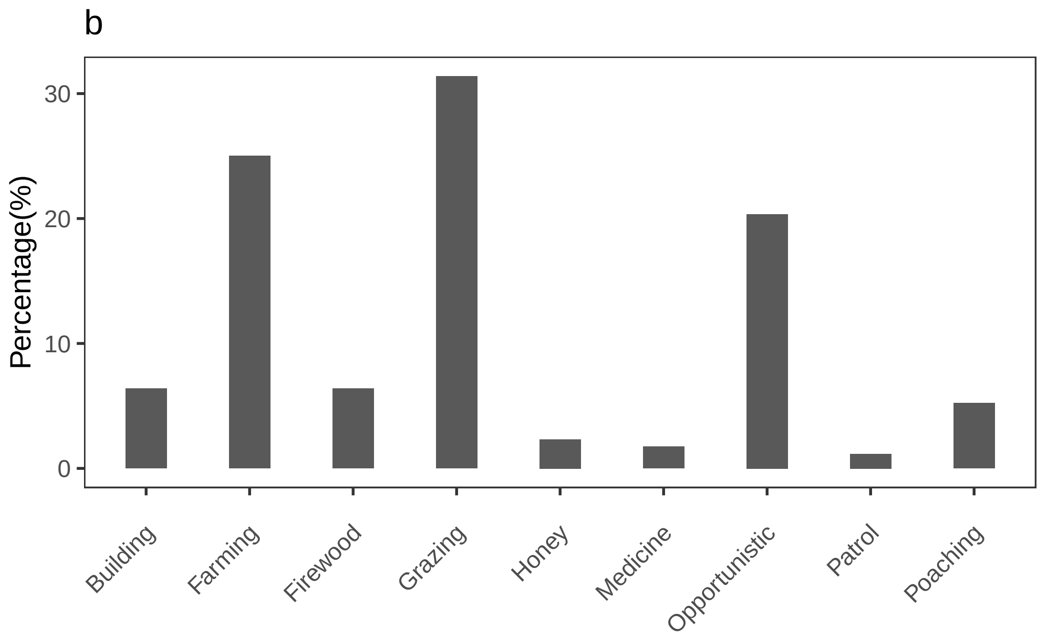


```
#Combine plot p1 &p2
grid.arrange(p1,p2,ncol=2)
```

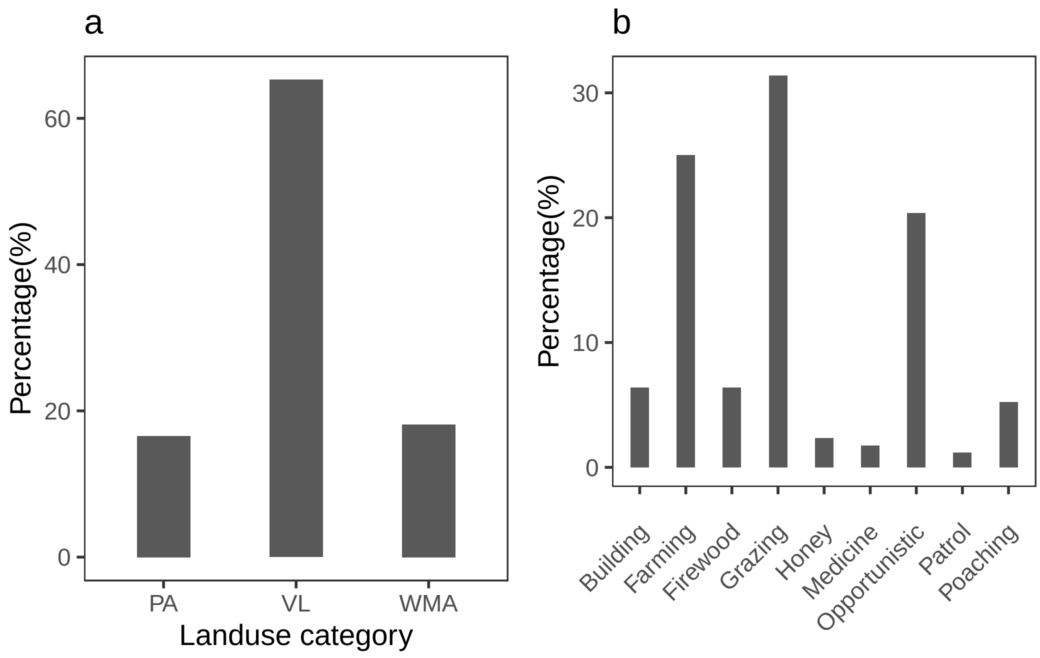


```
## WE WANT TO ANALYSE BY CATEGORIZING THE ACTIVITY TYPES INTO FOUR CATEGORIES
activity$prop <- activity$Percentage/100
sum(activity$prop)
```

```
[1] 1
```

```r
# patrol and poaching (rows 1 and 9)
# Opportunistic and medicine by themselves (Rows 7 and 8)
# Livelihood are the rest (rows 2 through 6)
## restructure the data
livelihood <- activity[c(2:6),]
pandp <- activity[c(1, 9),]
act_sub <- activity[c(7, 8),]

P_And_P <- data.frame(Activity = "Patrol_Poaching",
                      Count = sum(pandp$Count),
                      Percentage = sum(pandp$Percentage),
                      prop = sum(pandp$prop))
Livelihood <- data.frame(Activity = "Livelihood",
                         Count = sum(livelihood$Count),
                         Percentage = sum(livelihood$Percentage),
                         prop = sum(livelihood$prop)
)
fulldat2 <- bind_rows(Livelihood, P_And_P, act_sub)

activity_reduced <- chisq.test(fulldat2$Count)
activity_reduced
```

```
    Chi-squared test for given probabilities

data:  fulldat2$Count
X-squared = 211, df = 3, p-value <2e-16
```

```r
## X-squared = 211, df = 3, p-value <2e-16
## so at least one activity makes an individual more likely to encounter pangolins

Activity_reduced <-ggplot(fulldat2, aes(Activity, prop)) +
  geom_bar(position = position_dodge(0.5), stat = "identity", width = 0.4) +
  theme_bw(base_size = 11) +
  theme(panel.grid.major = element_blank(),
        panel.grid.minor = element_blank()) +
  ylab("Proportion")+xlab(NULL) +
  theme(axis.text.x = element_text(angle = 45, vjust = 0.8, hjust=1))
Activity_reduced
```

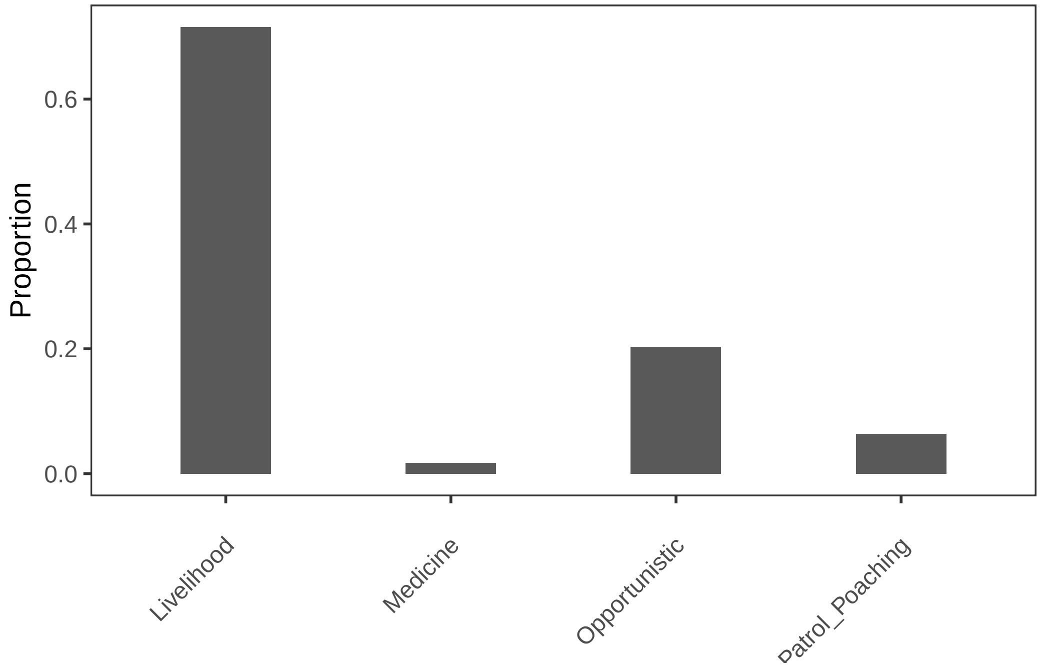


```
Activity_fig <- Activity_reduced + geom_text(aes(label = Count),
                                        vjust = 1, colour = "white")
```

Next is the habitat types (pangolin perspective) where the encounter occurred

```
#Let's perform chi-square test - this is testing for differences in habitat types where pang
fcount3<-matrix(c(24,11,33,12,19))
f_chisq3<-chisq.test(fcount3)
f_chisq3
```

```
    Chi-squared test for given probabilities

data:  fcount3
X-squared = 17, df = 4, p-value = 0.002
```

```
#Frequency of encountering pangolin btn habitat levels
habitat<-read_excel("Habitat.xlsx")
names(habitat)
```

```
[1] "Habitat"    "Counts"     "Percentage"
```

```
p7<-ggplot(habitat, aes(Habitat, Percentage))+
  geom_bar(stat = "identity", width = 0.4)+theme_bw(base_size = 11)+theme(panel.grid.major =
p7
```

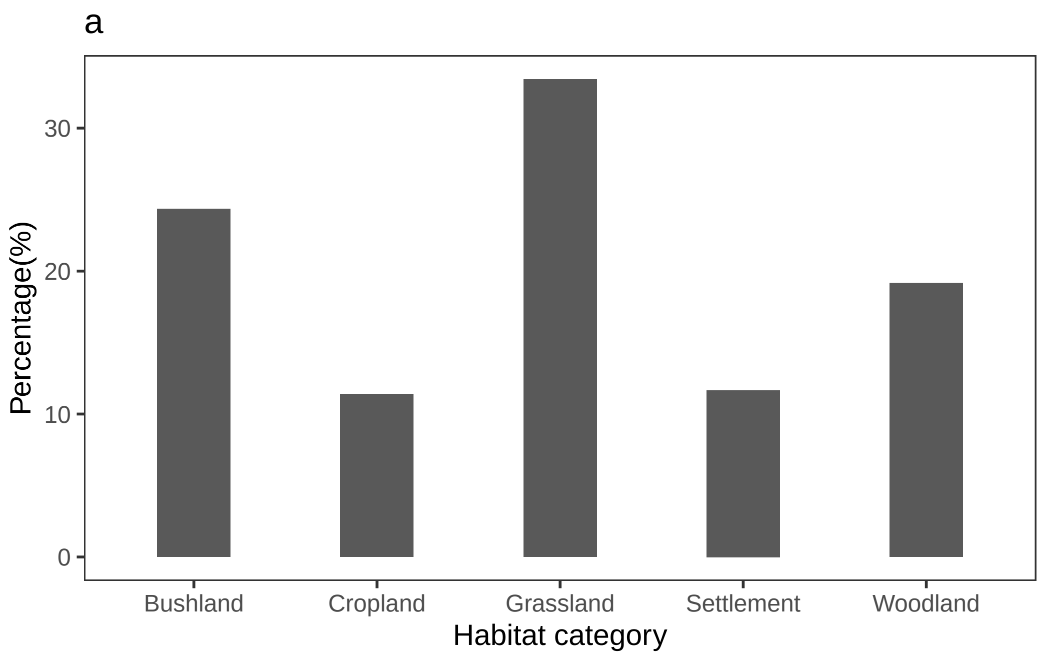

Explore correlations between the demographic predictor variables in the dataset

```
### CORRELATION MATRIX
str(mydata)
```

```
tibble [386 x 22] (S3: tbl_df/tbl/data.frame)
 $ Village          : chr [1:386] "Tungamalenga" "Tungamalenga" "Tungamalenga" "Tungamalenga"
 $ Tribe            : num [1:386] 2 5 8 8 8 8 5 8 5 5 ...
 $ Gender           : num [1:386] 1 1 1 1 1 1 1 1 1 1 ...
 $ Age              : num [1:386] 34 32 32 32 60 45 28 27 31 29 ...
 $ Occupation       : chr [1:386] "Agropastoralist" "Patoralist" "Patoralist" "Patoralist" ..
 $ Annual_Income    : num [1:386] 1500000 180000 4200000 4200000 200000 2000000 800000 100000
 $ Rounded_inc      : num [1:386] 1.5 0.18 4.2 4.2 0.2 2 0.8 1 2 0.4 ...
 $ Residence_years : num [1:386] 17 32 32 32 10 26 28 27 20 29 ...
 $ Education        : num [1:386] 2 2 3 3 1 1 3 2 2 3 ...
 $ Area_encountered: chr [1:386] "WMA" "VL" "PA" "PA" ...
 $ Habitat          : num [1:386] 1 3 5 5 3 3 4 3 3 3 ...
 $ Habitat_visit    : num [1:386] 26 52 52 52 52 52 52 52 1 52 ...
 $ Sighting_freq    : num [1:386] 1 1 1 1 1 1 1 1 1 1 ...
 $ Group_size       : num [1:386] 1 1 2 2 0 1 1 1 0 0 ...
```

```
 $ Hours            : POSIXct[1:386], format: "1899-12-31 08:00:00" "1899-12-31 17:00:00" ...
 $ Season           : num [1:386] 1 3 4 4 NA 4 4 4 NA NA ...
 $ Trend            : num [1:386] 3 2 2 2 NA 2 1 2 NA NA ...
 $ Vland            : num [1:386] 0 1 0 0 0 1 0 1 0 0 ...
 $ Area_VL          : num [1:386] NA 3 NA NA NA 3 NA 5 NA NA ...
 $ Activity         : chr [1:386] NA "Grazing" NA NA ...
 $ Landuse_effect   : num [1:386] 0 1 1 1 0 1 1 0 1 1 ...
 $ Reason           : num [1:386] 1 1 2 2 1 3 5 1 2 5 ...
```

```r
dat <- as.data.frame(unclass(mydata), stringsAsFactors = TRUE)
str(dat)
```

```
'data.frame':   386 obs. of  22 variables:
 $ Village          : Factor w/ 21 levels "Idodi","Igava",..: 21 21 21 21 21 21 21 21 9 9 ...
 $ Tribe            : num  2 5 8 8 8 8 5 8 5 5 ...
 $ Gender           : num  1 1 1 1 1 1 1 1 1 1 ...
 $ Age              : num  34 32 32 32 60 45 28 27 31 29 ...
 $ Occupation       : Factor w/ 5 levels "Agropastoralist",..: 1 5 5 5 5 5 3 5 3 3 ...
 $ Annual_Income    : num  1500000 180000 4200000 4200000 200000 2000000 800000 1000000 20000
 $ Rounded_inc      : num  1.5 0.18 4.2 4.2 0.2 2 0.8 1 2 0.4 ...
 $ Residence_years  : num  17 32 32 32 10 26 28 27 20 29 ...
 $ Education        : num  2 2 3 3 1 1 3 2 2 3 ...
 $ Area_encountered: Factor w/ 3 levels "PA","VL","WMA": 3 2 1 1 2 2 1 2 3 2 ...
 $ Habitat          : num  1 3 5 5 3 3 4 3 3 3 ...
 $ Habitat_visit    : num  26 52 52 52 52 52 52 52 1 52 ...
 $ Sighting_freq    : num  1 1 1 1 1 1 1 1 1 1 ...
 $ Group_size       : num  1 1 2 2 0 1 1 1 0 0 ...
 $ Hours            : POSIXct, format: "1899-12-31 08:00:00" "1899-12-31 17:00:00" ...
 $ Season           : num  1 3 4 4 NA 4 4 4 NA NA ...
 $ Trend            : num  3 2 2 2 NA 2 1 2 NA NA ...
 $ Vland            : num  0 1 0 0 0 1 0 1 0 0 ...
 $ Area_VL          : num  NA 3 NA NA NA 3 NA 5 NA NA ...
 $ Activity         : Factor w/ 9 levels "Building","Farming",..: NA 4 NA NA NA 4 NA 7 NA NA
 $ Landuse_effect   : num  0 1 1 1 0 1 1 0 1 1 ...
 $ Reason           : num  1 1 2 2 1 3 5 1 2 5 ...
```

```r
dat2 <- dat[, c(2,5:7, 10:22)]

model.matrix(~ 0+., data=dat2) %>%
  cor(use="pairwise.complete.obs") %>%
  ggcorrplot(show.diag = F, type="lower", lab=TRUE, lab_size=2)
```

```
Warning in cor(., use = "pairwise.complete.obs"): the standard deviation is
zero
```

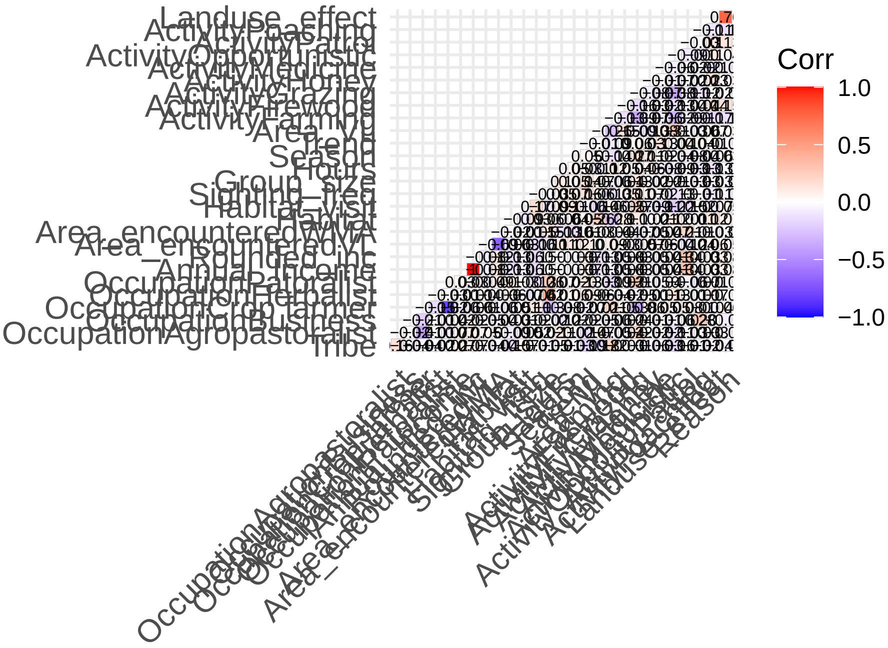


```
## there does not appear to be any high correlation between occupation and tribe. However, s

## for a further check for highly correlated variables, reduce the dataset and re-run the te
## remove columns:
dat3 <- dat[, c(2, 5, 6, 10:14, 16:22)]
model.matrix(~0+., data=dat3) %>%
  cor(use="pairwise.complete.obs") %>%
  ggcorrplot(show.diag = F, type="lower", lab=TRUE, lab_size=2)
```

```
Warning in cor(., use = "pairwise.complete.obs"): the standard deviation is
zero
```

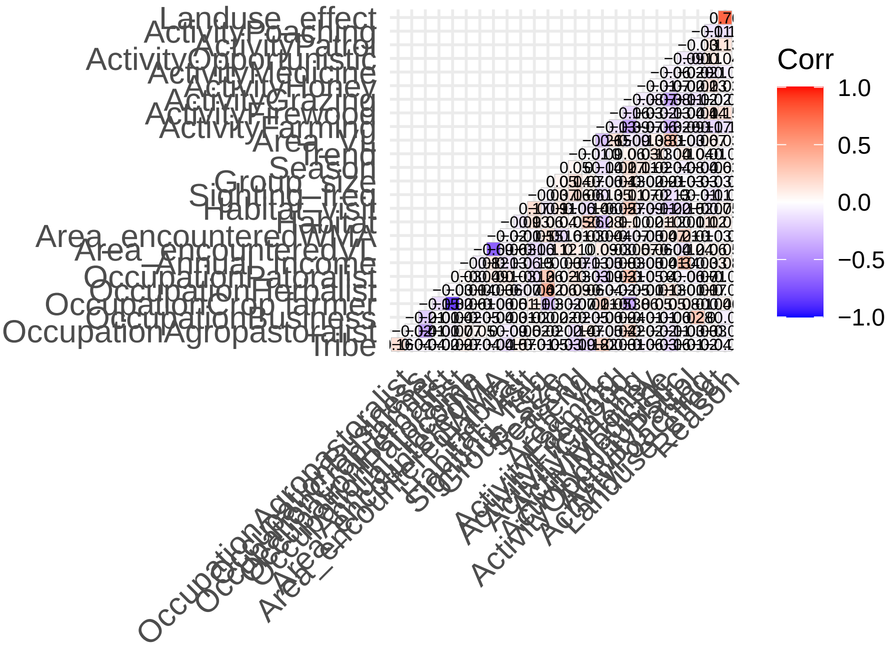


```
# should also check un-factored "Tribe" with the correlations!
dat4 <- dat[, c("Tribe", "Gender", "Age", "Occupation", "Rounded_inc",
               "Residence_years", "Education")]
str(dat4)
```

```
'data.frame':   386 obs. of  7 variables:
 $ Tribe          : num  2 5 8 8 8 8 5 8 5 5 ...
 $ Gender         : num  1 1 1 1 1 1 1 1 1 1 ...
 $ Age            : num  34 32 32 32 60 45 28 27 31 29 ...
 $ Occupation     : Factor w/ 5 levels "Agropastoralist",..: 1 5 5 5 5 5 3 5 3 3 ...
 $ Rounded_inc    : num  1.5 0.18 4.2 4.2 0.2 2 0.8 1 2 0.4 ...
 $ Residence_years: num  17 32 32 32 10 26 28 27 20 29 ...
 $ Education      : num  2 2 3 3 1 1 3 2 2 3 ...
```

```
model.matrix(~0+., data=dat4) %>%
  cor(use="pairwise.complete.obs") %>%
  ggcorrplot(show.diag = F, type="lower", lab=TRUE, lab_size=2)
```

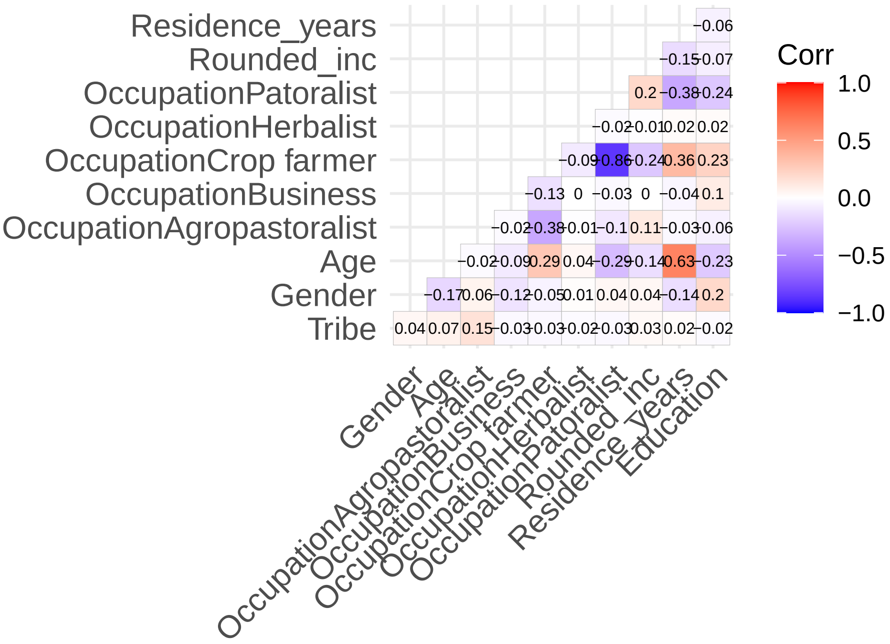


```
## For strong correlations (greater than 0.5 or less than -0.5) Crop Farmer is negatively co

## THE ABOVE CORRELATION CHECKS ARE TREATING SEVERAL FACTOR VARIABLES AS NUMERIC
## Occupation is being treated as a factor, but (amongst demographic variables), sex, educat
## change tribe to a factor and check for correlations
## JUST THE TRIBES
dat5 <- dat4
dat5$Tribe <- as.factor(dat5$Tribe)
model.matrix(~0+., data=dat5) %>%
  cor(use="pairwise.complete.obs") %>%
  ggcorrplot(show.diag = F, type="lower", lab=TRUE, lab_size=2)
```

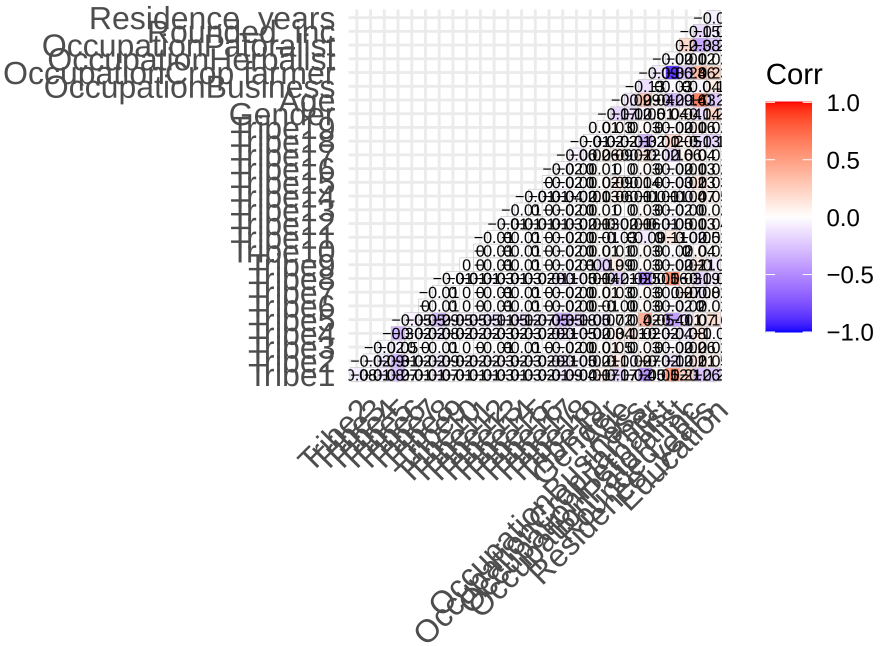


```r
## TRIBE AND SEX
dat6 <- dat5
dat6$Gender <- as.factor(dat6$Gender)
model.matrix(~0+., data=dat6) %>%
  cor(use="pairwise.complete.obs") %>%
  ggcorrplot(show.diag = F, type="lower", lab=TRUE, lab_size=2)
```

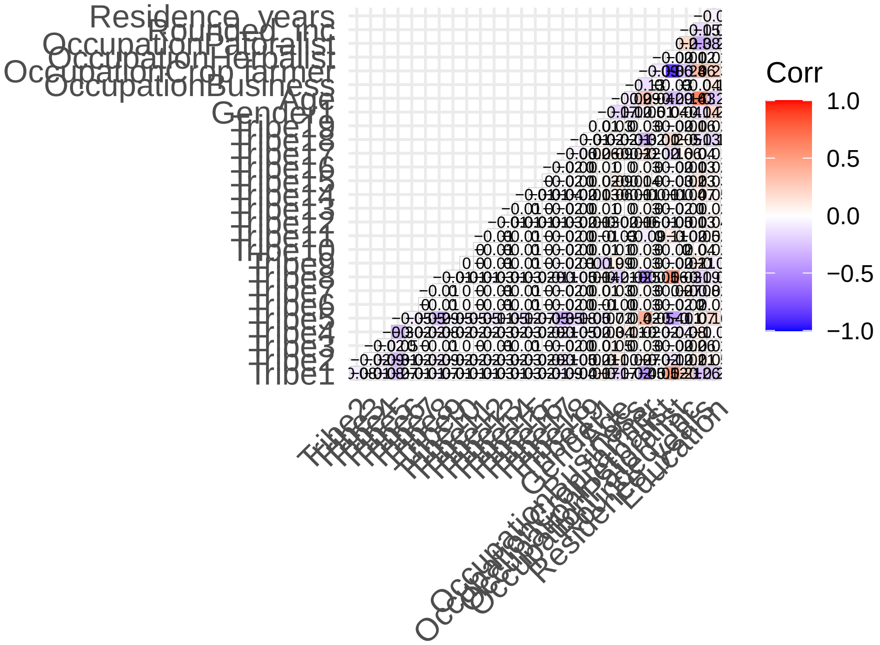

```
## TRIBE, SEX, and EDUCATION
dat7 <- dat6
dat7$Education <- as.factor(dat7$Education)
model.matrix(~0+., data=dat7) %>%
  cor(use="pairwise.complete.obs") %>%
  ggcorrplot(show.diag = F, type="lower", lab=TRUE, lab_size=2)
```

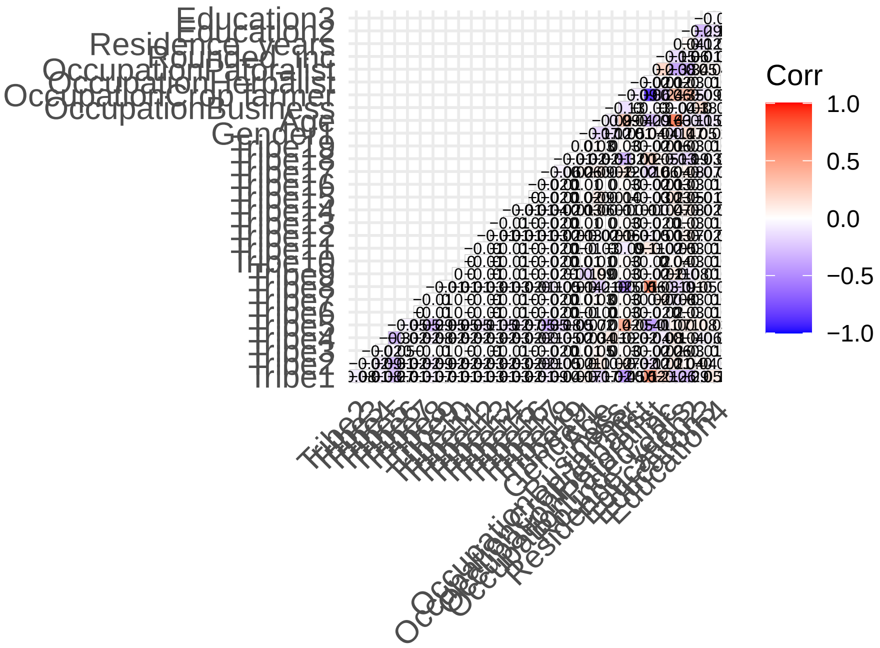


```
## demographic variables in the first regression: Tribe, Gender, Age, Occupation, Rounded_in
```

Summarize Encounters and Activity by Tribe

```
## using the factors for Tribe, do a column summary for Encounters and Tribe. We need to gro
count(mydata, Tribe) ## overall counts
```

```
# A tibble: 19 x 2
   Tribe     n
   <dbl> <int>
 1     1    24
 2     2    32
 3     3     1
 4     4    31
 5     5   199
 6     6     1
 7     7     1
```

```
 8     8    29
 9     9     1
10    10     1
11    11     1
12    12     4
13    13     1
14    14     5
15    15     2
16    16     1
17    17    40
18    18    11
19    19     1
```

```r
target <- c(1, 2, 4, 5, 8, 17, 18) ## which Tribe factors have sufficient sample size
dat_t <- mydata %>% ## these are the Tribes with sufficient sample sizes
  filter(
    Tribe %in% target
  )
dat_n <- anti_join(mydata, dat_t) ## these are the Tribes that have to be grouped together
```

```
Joining with `by = join_by(Village, Tribe, Gender, Age, Occupation,
Annual_Income, Rounded_inc, Residence_years, Education, Area_encountered,
Habitat, Habitat_visit, Sighting_freq, Group_size, Hours, Season, Trend, Vland,
Area_VL, Activity, Landuse_effect, Reason)`
```

```r
Other <- data.frame(Tribe = factor(), ## initialize the dataframe
                    Encounters = integer(),
                    Total_count = integer()

)
Total_count <- nrow(dat_n) ## calculate the different categories for the grouped Tribes
Encounters <- sum(dat_n$Vland)
Tribe <- 20
Other <- data.frame(Tribe, Encounters, Total_count)

Large_n <- dat_t %>% ## summarize the Tribes with larger sample sizes
    group_by(Tribe) %>%
      summarise(
        Encounters = sum(Vland),
        Total_count = n()
      )
fulldat <- bind_rows(Large_n, Other) ## combine all Tribes into a single dataframe
fulldat$prop <- fulldat$Encounters/fulldat$Total_count ## Calculate proportions of survey re
```

```r
## test for significant differences in probability of encountering a pangolin by Tribe or Gr
Tribe_Test <- prop.test(fulldat$Encounters, fulldat$Total_count)
```

```
Warning in prop.test(fulldat$Encounters, fulldat$Total_count): Chi-squared
approximation may be incorrect
```

```r
Tribe_Test
```

```
    8-sample test for equality of proportions without continuity correction

data:  fulldat$Encounters out of fulldat$Total_count
X-squared = 11, df = 7, p-value = 0.1
alternative hypothesis: two.sided
sample estimates:
prop 1 prop 2 prop 3 prop 4 prop 5 prop 6 prop 7 prop 8
 0.417  0.719  0.613  0.633  0.552  0.500  0.455  0.450
```

```r
# No group is statistically more likely to encounter pangolins: X-squared = 10.748, df = 7,

#Let's perform chi-square test regarding if the proportion of reported pangolin encounters w
fulldat$propoftotal <- fulldat$Encounters/sum(fulldat$Encounters)
fulldat$pctoftotal <- fulldat$propoftotal*100
tribechi <- as.matrix(fulldat$pctoftotal)
f_chisq4<-chisq.test(tribechi)
## THIS TEST IS SIGNIFICANT but the results may be skewed by the sample sizes of some ethnic


## make a figure
str(fulldat)
```

```
tibble [8 x 6] (S3: tbl_df/tbl/data.frame)
 $ Tribe      : num [1:8] 1 2 4 5 8 17 18 20
 $ Encounters : num [1:8] 10 23 19 126 16 20 5 9
 $ Total_count: int [1:8] 24 32 31 199 29 40 11 20
 $ prop       : num [1:8] 0.417 0.719 0.613 0.633 0.552 ...
 $ propoftotal: num [1:8] 0.0439 0.1009 0.0833 0.5526 0.0702 ...
 $ pctoftotal : num [1:8] 4.39 10.09 8.33 55.26 7.02 ...
```

```r
fulldat$Tribe <- as.factor(fulldat$Tribe)
Tribe_prop <-ggplot(fulldat, aes(Tribe, prop)) +
  geom_bar(position = position_dodge(0.5), stat = "identity", width = 0.4) +
```

```r
  theme_bw(base_size = 11) +
  theme(panel.grid.major = element_blank(),
        panel.grid.minor = element_blank()) +
  ylab("Proportion")+xlab("Tribe Factor")
Tribe_fig <- Tribe_prop + geom_text(aes(label = Total_count),
                       vjust = 1.5, colour = "white")
```

Make a figure to include the four chsquared tests as subfigures

```r
Lmgmt_fig <-ggplot(landuse, aes(Landuse, Percentage)) +
  geom_bar(stat = "identity", width = 0.4) +
  theme_bw(base_size = 11) +
  theme(panel.grid.major = element_blank(),
        panel.grid.minor = element_blank()) +
  ylab("Percentage(%)") +
  xlab("Landuse category")+ggtitle("a")
# Lmgmt_fig


Activity_fig2 <- Activity_reduced +
  geom_text(aes(label = Count), vjust = 1, colour = "white") +
  ggtitle("b")

hab_fig <- p7 +
  ggtitle("c")

Tribe_fig2 <- Tribe_fig +
  ggtitle("d")

grid.arrange(Lmgmt_fig, Activity_fig2, hab_fig, Tribe_fig2, ncol = 2)
```

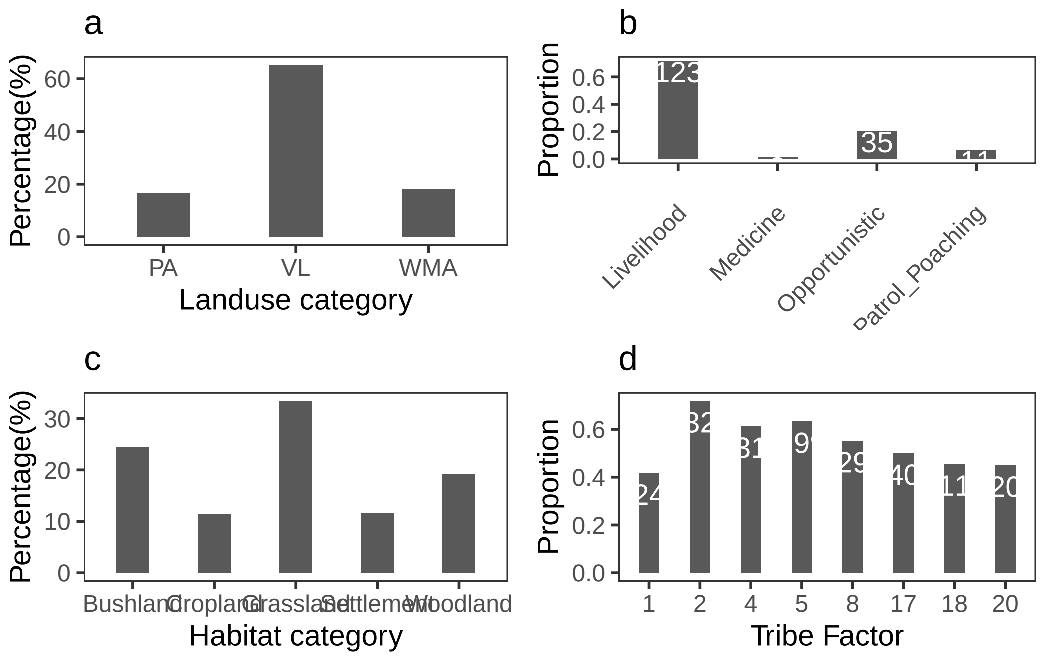

Next research question - do any demographic variables predict if someone would encounter pangolins? Including Tribe as a factor would add 19 variables to the results. Adding gender and education as factors would add one and three variables, respectively.

```
#Do demographic variables influence encountering of
#pangolins in the village land?
#Perform logistic regression
## AS DISCOVERED - age and residence years are correlated (R^2 > 0.6) . Which one should be

## change gender and education to factors
mydata$Gender <- as.factor(mydata$Gender)
mydata <- mydata %>%
  mutate(
    sex = if_else(Gender == 0, "F", "M")
  )
mydata$Education <- as.factor((mydata$Education))
mydata$sex <- as.factor(mydata$sex)
## Since the correlation is R^2 = 0.63, keep both age and residence years in the model, with
m1_ageandres <- glm(Vland~sex+Age+Occupation+Rounded_inc+Residence_years+Education,
        data=mydata, family = binomial(link = logit))
summary(m1_ageandres)
```

```
Call:
glm(formula = Vland ~ sex + Age + Occupation + Rounded_inc +
```

```
    Residence_years + Education, family = binomial(link = logit),
    data = mydata)

Coefficients:
                       Estimate Std. Error z value Pr(>|z|)
(Intercept)            -0.51529    0.82102   -0.63    0.530
sexM                    0.31210    0.43749    0.71    0.476
Age                     0.00788    0.00863    0.91    0.361
OccupationBusiness     14.71819  623.65592    0.02    0.981
OccupationCrop farmer   0.24263    0.53262    0.46    0.649
OccupationHerbalist    14.20192  882.74354    0.02    0.987
OccupationPatoralist    0.06738    0.57055    0.12    0.906
Rounded_inc            -0.04241    0.02387   -1.78    0.076 .
Residence_years        -0.00306    0.00733   -0.42    0.677
Education2              0.26363    0.27197    0.97    0.332
Education3              0.04174    0.61158    0.07    0.946
Education4              0.56349    1.25573    0.45    0.654
---
Signif. codes:  0 '***' 0.001 '**' 0.01 '*' 0.05 '.' 0.1 ' ' 1

(Dispersion parameter for binomial family taken to be 1)

    Null deviance: 522.34  on 385  degrees of freedom
Residual deviance: 510.10  on 374  degrees of freedom
AIC: 534.1

Number of Fisher Scoring iterations: 13
```

Next is a stepwise regression to find the optimal model of demographic variables that predict encountering pangolins, and a figure for the significant variables.

```
#Use stepAIC to determine final model for demmographic variables that affect pangolin encoun

m2<-stepAIC(m1_ageandres)
```

```
Start:  AIC=534
Vland ~ sex + Age + Occupation + Rounded_inc + Residence_years +
    Education

                  Df Deviance AIC
- Education        3      511 529
- Occupation       4      514 530
- Residence_years  1      510 532
- sex              1      511 533
```

```
- Age              1      511 533
<none>                    510 534
- Rounded_inc      1      514 536

Step:  AIC=529
Vland ~ sex + Age + Occupation + Rounded_inc + Residence_years

                  Df Deviance AIC
- Occupation       4      516 526
- Residence_years  1      511 527
- Age              1      512 528
- sex              1      512 528
<none>                    511 529
- Rounded_inc      1      515 531

Step:  AIC=526
Vland ~ sex + Age + Rounded_inc + Residence_years

                  Df Deviance AIC
- Residence_years  1      516 524
- Age              1      516 524
- sex              1      517 525
<none>                    516 526
- Rounded_inc      1      521 529

Step:  AIC=524
Vland ~ sex + Age + Rounded_inc

              Df Deviance AIC
- sex          1      517 523
- Age          1      517 523
<none>                516 524
- Rounded_inc  1      521 527

Step:  AIC=523
Vland ~ Age + Rounded_inc

              Df Deviance AIC
- Age          1      517 521
<none>                517 523
- Rounded_inc  1      521 525

Step:  AIC=521
Vland ~ Rounded_inc
```

```
              Df Deviance AIC
<none>                517 521
- Rounded_inc  1      522 524
```

```r
summary(m2)
```

```

Call:
glm(formula = Vland ~ Rounded_inc, family = binomial(link = logit),
    data = mydata)

Coefficients:
            Estimate Std. Error z value Pr(>|z|)
(Intercept)   0.4959     0.1193    4.16  3.3e-05 ***
Rounded_inc  -0.0494     0.0233   -2.12    0.034 *
---
Signif. codes:  0 '***' 0.001 '**' 0.01 '*' 0.05 '.' 0.1 ' ' 1

(Dispersion parameter for binomial family taken to be 1)

    Null deviance: 522.34  on 385  degrees of freedom
Residual deviance: 517.06  on 384  degrees of freedom
AIC: 521.1

Number of Fisher Scoring iterations: 4
```

```r
#Plot a graph for income
names(mydata)
```

```
 [1] "Village"          "Tribe"            "Gender"           "Age"
 [5] "Occupation"       "Annual_Income"    "Rounded_inc"      "Residence_years"
 [9] "Education"        "Area_encountered" "Habitat"          "Habitat_visit"
[13] "Sighting_freq"    "Group_size"       "Hours"            "Season"
[17] "Trend"            "Vland"            "Area_VL"          "Activity"
[21] "Landuse_effect"   "Reason"           "sex"
```

```r
p3<-ggplot(mydata, aes(Rounded_inc,Vland))+ylab("Encountering probability")+ggtitle("a")+
  xlab("Annual income(x1 million)")+geom_point()+theme_classic(base_size = 12)+
  geom_smooth(method = "glm",method.args=list(family="binomial"), se=FALSE)
p3
```

```
`geom_smooth()` using formula = 'y ~ x'
```

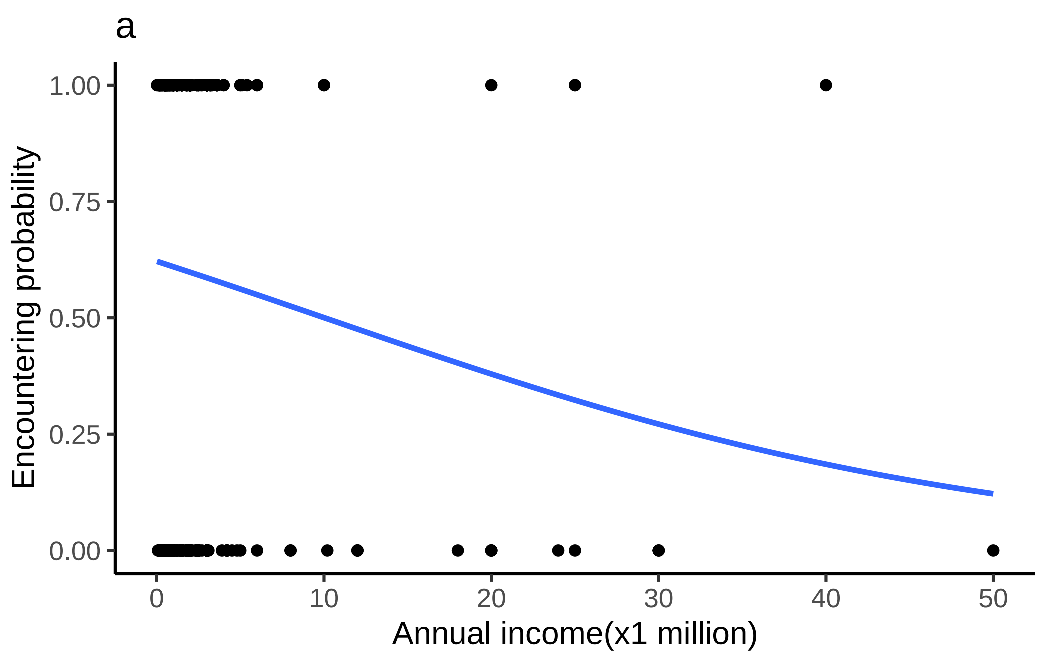

Analysis of group size

```
#What is the mean and standard deviation for pangolin group size?
mean(mydata$Group_size)
```

```
[1] 0.907
```

```
sd(mydata$Group_size)
```

```
[1] 0.541
```

```
#Import all data without NA
mydata2<-read_excel("Data3.xlsx")

###############################
## Poisson GLM for encountered pangolin group size in relation to land use and human activit

names(mydata2)
```

```
 [1] "Village"          "Tribe"             "Gender"          "Age"
 [5] "Occupation"       "Annual_Income"     "Rounded_inc"     "Residence_years"
 [9] "Education"        "Area_encountered"  "Habitat"         "Habitat_visit"
[13] "Sighting_freq"    "Group_size"        "Hours"           "Season"
[17] "Trend"            "Vland"             "Area_VL"         "Activity"
[21] "Landuse_effect"   "Reason"
```

```r
## need new factor column to account for the categories of activity types
# patrol and poaching (rows 1 and 9)
# Opportunistic and medicine by themselves (Rows 7 and 8)
# Livelihood are the rest (rows 2 through 6)
unique(mydata2$Activity)
```

```
[1] "Grazing"       "Opportunistic" "Building"      "Poaching"
[5] "Farming"       "Honey"         "Firewood"      "Medicine"
[9] "Patrol"
```

```r
mydata2 <- mydata2 %>%
  mutate(
    activity_cate = case_when(
      str_detect(Activity, "Opportunistic") ~ "Opportunistic",
      str_detect(Activity, "Medicine") ~ "Medicine",
      str_detect(Activity, "Patrol") | str_detect(Activity, "Poaching") ~ "Patrol and Poachi
      TRUE ~ "Livelihood"
    )
  )
mydata2$activity_cate <- as.factor(mydata2$activity_cate)
# quasi-poisson for zero inflation
m4<-glm(Group_size~Area_encountered+activity_cate, data = mydata2, family = quasipoisson(lin
summary(m4)
```

```
Call:
glm(formula = Group_size ~ Area_encountered + activity_cate,
    family = quasipoisson(link = log), data = mydata2)

Coefficients:
                                 Estimate Std. Error t value Pr(>|t|)
(Intercept)                        0.0654     0.1138    0.58    0.566
Area_encounteredVL                -0.1543     0.1213   -1.27    0.205
Area_encounteredWMA                0.1540     0.1474    1.05    0.297
activity_cateMedicine             -0.6907     0.4070   -1.70    0.091 .
activity_cateOpportunistic         0.0682     0.0921    0.74    0.460
activity_catePatrol and Poaching  -0.3928     0.1804   -2.18    0.031 *
---
Signif. codes:  0 '***' 0.001 '**' 0.01 '*' 0.05 '.' 0.1 ' ' 1

(Dispersion parameter for quasipoisson family taken to be 0.326)

    Null deviance: 83.869  on 227  degrees of freedom
```

```
Residual deviance: 78.000  on 222  degrees of freedom
AIC: NA

Number of Fisher Scoring iterations: 5
```

```
sum_1<-plyr::ddply(mydata2,c("Area_encountered"),summarise,
                        N=length(Group_size),
                        mean_res=mean(Group_size,na.rm = T),
                        sd_res=sd(Group_size,na.rm = T),
                        sed_res=sd_res/sqrt(N))
sum_1
```

```
  Area_encountered   N mean_res sd_res sed_res
1               PA  24    1.083  0.408  0.0833
2               VL 172    0.901  0.492  0.0375
3              WMA  32    1.188  0.998  0.1764
```

```
p5<-ggplot2::ggplot(sum_1,aes(x=Area_encountered,y=mean_res))+
  geom_bar(stat = "identity", width = 0.4)+ylab("Mean group size")+geom_errorbar(aes(ymin=me
  theme_bw()+xlab("Land use category")+
  theme(panel.grid.major = element_blank(), panel.grid.minor = element_blank())

p5
```

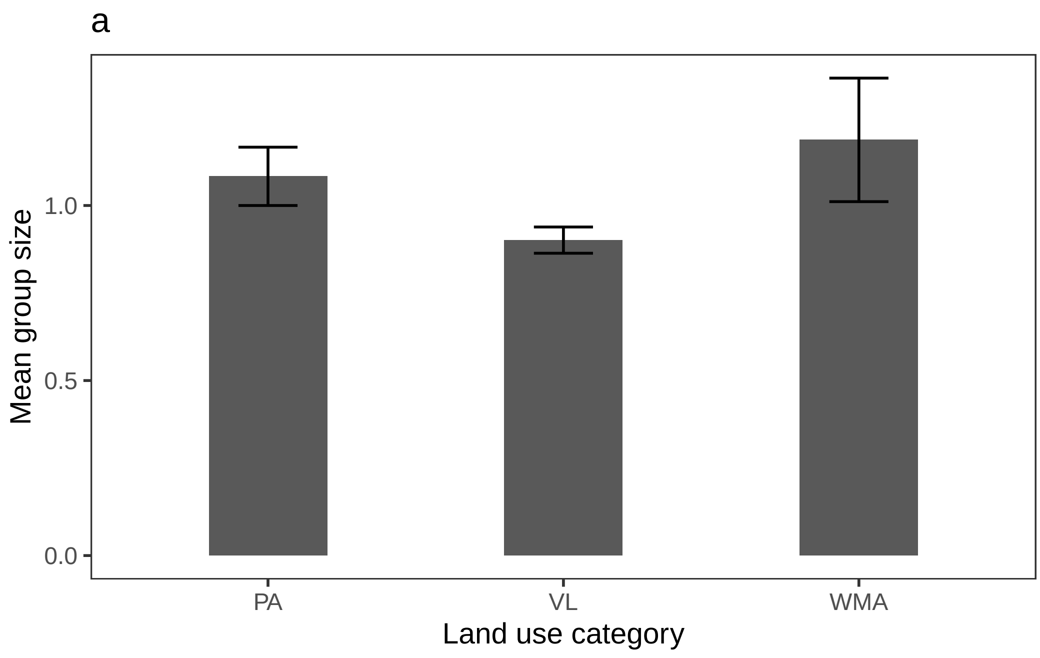

```
#Crete a plot for human activity
sum_2<-plyr::ddply(mydata2,c("Activity"),summarise,
                   N=length(Group_size),
                   mean_res=mean(Group_size,na.rm = T),
                   sd_res=sd(Group_size,na.rm = T),
                   sed_res=sd_res/sqrt(N))
sum_2
```

```
       Activity  N mean_res sd_res sed_res
1      Building 15    0.867  0.352  0.0909
2       Farming 54    0.981  0.363  0.0494
3      Firewood 13    0.769  0.439  0.1216
4       Grazing 73    1.027  0.781  0.0914
5         Honey  4    0.750  0.500  0.2500
6      Medicine  4    0.500  0.577  0.2887
7 Opportunistic 49    1.061  0.556  0.0794
8        Patrol  5    1.000  0.000  0.0000
9       Poaching 11    0.545  0.522  0.1575
```

```
p6<-ggplot2::ggplot(sum_2,aes(x=Activity,y=mean_res))+
  geom_bar(stat = "identity", width = 0.4)+ylab("Mean group size")+geom_errorbar(aes(ymin=me
  theme_bw()+xlab("")+
  theme(panel.grid.major = element_blank(), panel.grid.minor = element_blank())+theme(axis.t

p6
```

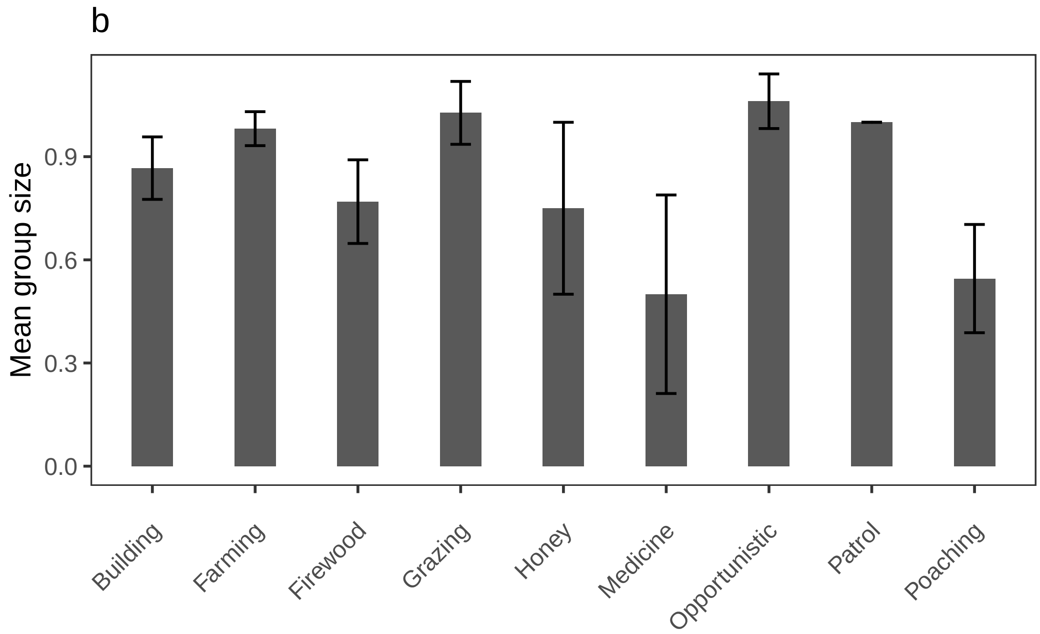

```
#Combine p5&p6
grid.arrange(p5,p6, ncol=2)
```

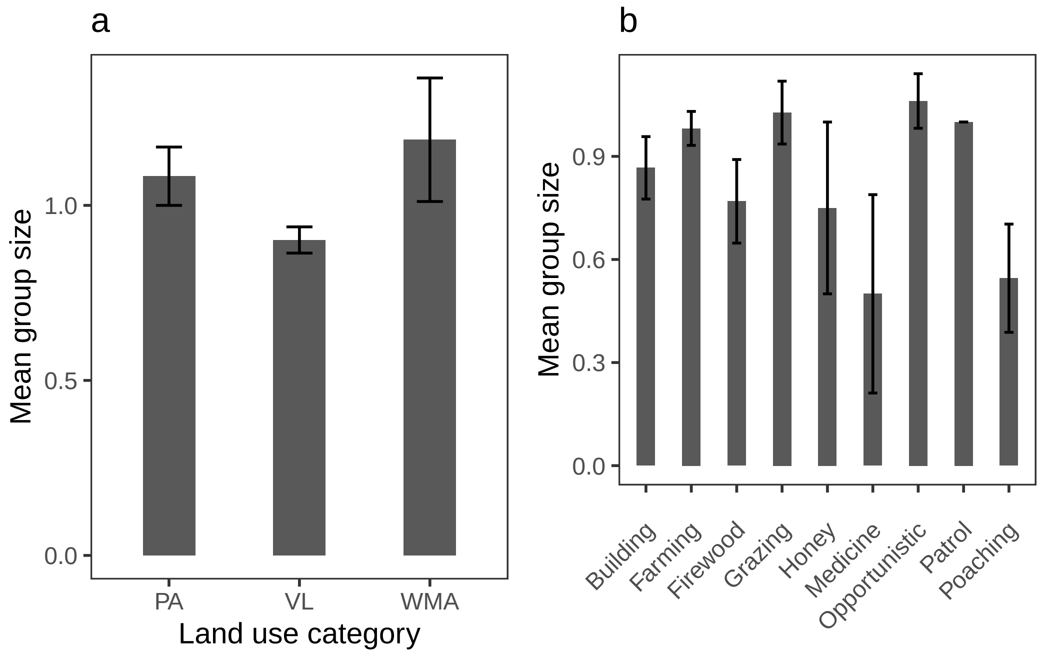

Next is the chi-squared test of perceived trend in pangolin encounters

```
################Assess trend of pangolin sightings over the past 5 years
xtabs(~Trend, data = mydata)
```

```
Trend
  1   2   3   4
145 125  39  18
```

```
# Trend
#   1   2   3   4
# 145 125  39  18

#Let's perform chi-square test for trend
fcount4<-matrix(c(44,38,12,6)) ## these are the percentages of responses in column "Trend" (
f_chisq4<-chisq.test(fcount4)
f_chisq4
```

```
    Chi-squared test for given probabilities
```

```
data:  fcount4
X-squared = 42, df = 3, p-value = 3e-09
```

```
# grid.arrange(p7,p8, ncol=2)
############################################
```

Activity pattern / time of day of pangolin encounters

```
#############################
## CHW Analysis ##
#############################
library(anytime)
library(lubridate)
```

```

Attaching package: 'lubridate'

The following objects are masked from 'package:base':

    date, intersect, setdiff, union
```

```
library(card)
```

```
Warning: package 'card' was built under R version 4.4.2
```

```
library(astroFns)
library(overlap)
```

```
Loading required package: suntools
```

```
library(activity)

dat <- dat %>%
  mutate(
    dt = ymd_hms(Hours),
   time = format(dt, format = "%H:%M:%S"),
   time2 = hms::as_hms(dt))
```

```
Warning: There was 1 warning in `mutate()`.
i In argument: `dt = ymd_hms(Hours)`.
Caused by warning:
!  30 failed to parse.
```

```r
activity_pattern <- dat %>%
  filter(
    !is.na(time2)
  ) %>%
  mutate(
    ## convert time to radians
timerad = hms2rad(time2) ## this is from the astroFns package
  )
attach(activity_pattern) ## use this command to keep from calling the df name in the followi
```

```
The following object is masked _by_ .GlobalEnv:

    Tribe
```

```r
result_pangolin <- fitact(timerad)  # Very basic fit without any adjustments or bootstrappin
# result_pangolin     # View the data, you can scroll up to the various slots of output info

# And plot the activity pattern
plot(result_pangolin)     # This is a very basic plot and defaults to frequency of observati
```

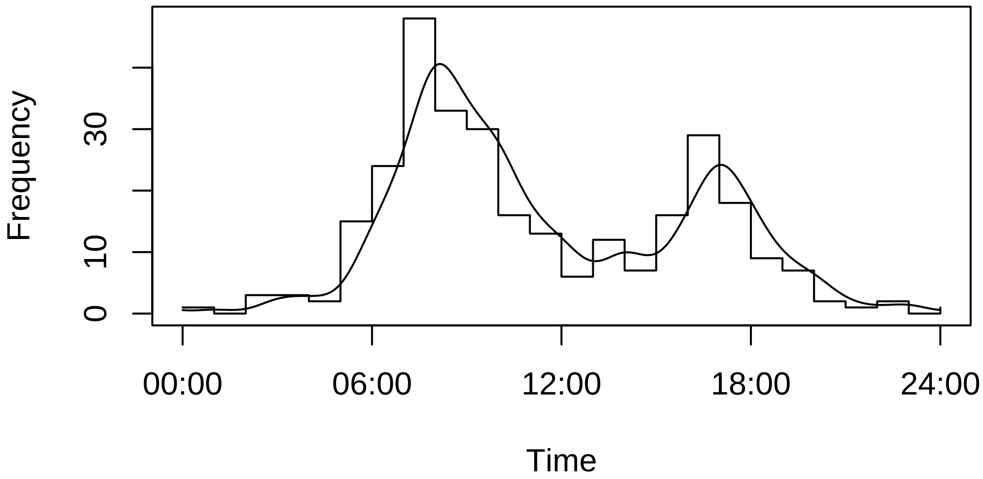


```r
# Some modifications

plot(result_pangolin, yunit = "density", data = "rug")   # Changed the background barplots t
```

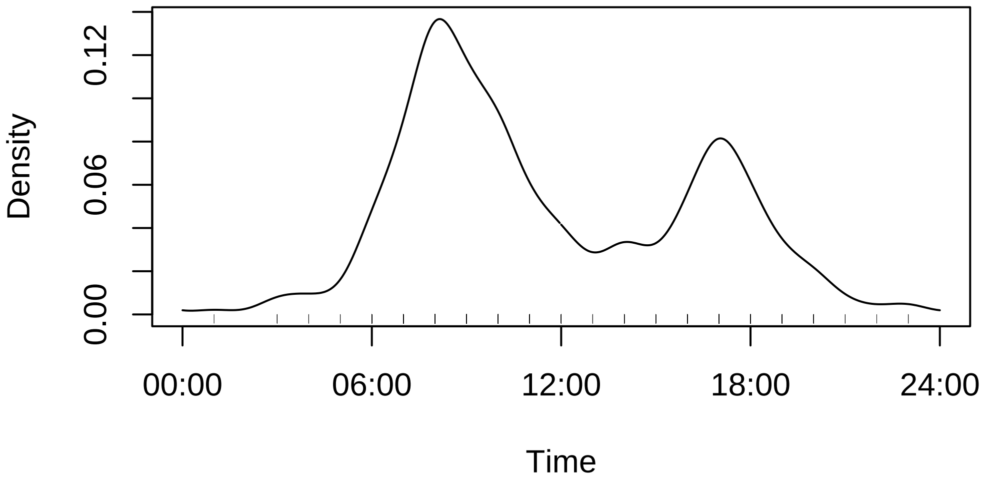


```
plot(result_pangolin, yunit = "density", data = "none")
```

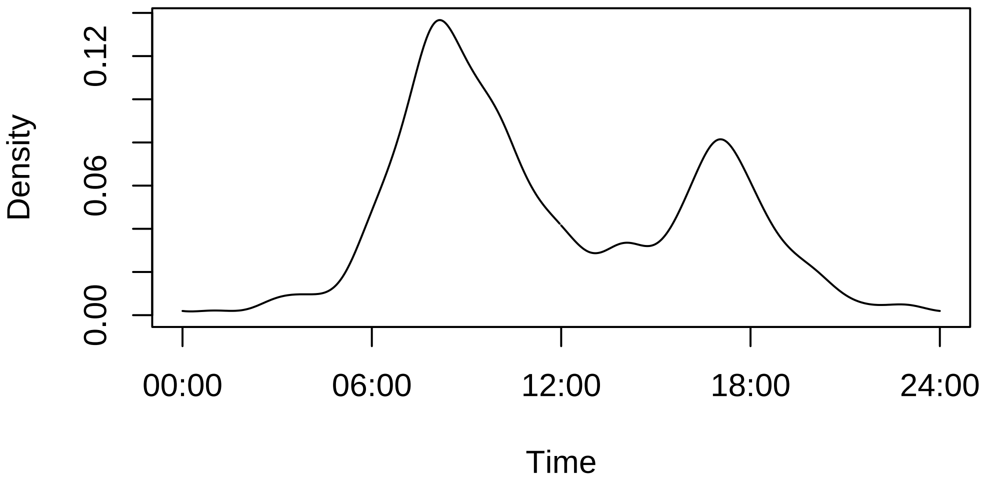


```
# create some confidence limits - we need to rerun the fitact function again and sample this
result_pangolin_CI <- fitact(timerad, sample = "data", reps = 100)  # Resamples from the dat
```

```
# Plot the resulting activity pattern and now include the confidence limits
plot(result_pangolin_CI, main="(a) Activity = 0.3048 RESULTS WITH CI",
     yunit = "density", cline=list(col="red", lty=3), data = "none")
```

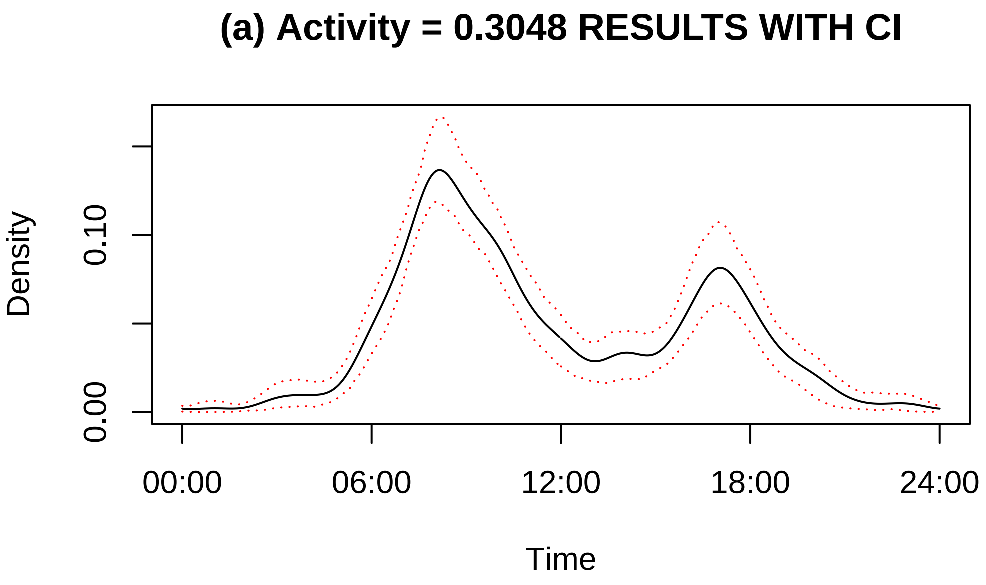


```
# But, does change in season impact the result - can use the "solartime" function to anchor
sol_times <- solartime(activity_pattern$time2, -7.7247, 35.6189, +3)    # Decimal degrees co-
str(sol_times)    # Results in a dataset with original time, clock time and solar time in rad
```

```
List of 3
 $ input: POSIXlt[1:297], format: "1970-01-01 08:00:00" "1970-01-01 17:00:00" ...
 $ clock: num [1:297] 2.09 4.45 2.09 2.09 4.45 ...
 $ solar: num [1:297] 2.09 4.45 2.09 2.09 4.45 ...
```

```
result_sol <- fitact(sol_times$solar)      # Activity fitted to solar time
result_sol                                 # Print the solar time
```

```
An object of class "actmod"
Slot "data":
  [1] 2.094 4.451 2.094 2.094 4.451 4.974 4.189 3.665 2.094 2.356 2.356 6.021
 [13] 3.927 4.189 1.047 3.927 2.094 4.712 4.712 2.094 1.833 3.142 2.880 2.356
 [25] 4.189 3.665 4.189 2.356 4.189 3.927 3.403 2.356 2.356 1.833 1.833 2.618
 [37] 4.712 1.833 4.189 2.356 4.451 4.974 4.712 1.571 2.094 2.094 2.356 3.142
 [49] 1.571 1.833 3.142 3.665 4.189 4.451 2.094 2.094 4.712 2.880 4.189 4.974
```

```
 [61] 5.498 2.094 2.356 1.047 2.880 1.833 4.451 2.094 2.618 4.451 2.094 2.356
 [73] 4.712 4.712 1.571 2.094 1.833 2.618 4.189 3.665 4.451 2.094 2.618 2.880
 [85] 3.142 2.618 3.927 2.356 4.712 3.665 2.094 2.880 3.142 1.833 4.712 2.618
 [97] 1.571 2.094 4.189 3.403 2.618 3.142 4.451 4.712 3.927 2.618 2.880 2.356
[109] 2.094 2.618 0.785 3.665 4.451 2.356 2.618 1.571 1.571 1.571 3.403 2.094
[121] 3.142 4.451 4.451 2.618 4.451 2.618 1.571 4.451 4.451 1.571 2.356 6.021
[133] 2.618 2.094 2.356 4.451 3.665 3.142 4.189 3.142 2.880 3.403 2.094 1.833
[145] 1.833 3.665 1.833 1.833 1.833 4.189 2.880 1.833 2.880 1.309 5.236 5.236
[157] 4.712 2.094 4.451 2.094 2.618 5.236 2.094 2.618 2.356 4.189 2.618 4.451
[169] 5.760 4.189 2.880 1.833 2.094 4.974 3.142 4.451 2.094 3.665 4.712 4.712
[181] 5.498 2.356 4.451 2.094 3.927 2.618 4.974 4.451 2.356 4.712 2.356 2.356
[193] 4.712 4.451 4.451 2.094 4.189 5.236 1.571 4.451 2.094 2.094 2.356 2.356
[205] 2.356 2.094 2.356 1.833 2.880 3.142 3.403 1.833 2.356 2.356 2.094 2.094
[217] 2.880 2.094 2.094 4.974 3.927 2.618 4.451 4.712 5.236 4.451 2.880 2.094
[229] 2.356 2.618 2.356 2.618 2.880 2.094 3.665 2.618 3.142 1.309 4.712 4.451
[241] 3.142 2.094 1.833 2.618 5.236 1.571 1.571 1.571 4.974 1.047 1.571 4.451
[253] 4.712 1.833 2.356 4.974 1.833 1.833 0.785 1.571 2.094 2.094 2.356 1.833
[265] 4.451 2.094 3.403 5.236 2.094 3.665 1.833 2.094 2.094 2.618 0.785 4.189
[277] 4.974 2.356 2.618 2.618 2.618 2.356 2.880 3.665 2.356 2.094 2.880 2.094
[289] 1.833 2.618 2.618 2.094 4.451 2.618 2.618 2.094 0.262

Slot "wt":
[1] 1

Slot "bw":
[1] 34

Slot "adj":
[1] 1

Slot "pdf":
           x       y
  [1,] 0.0000 0.00743
  [2,] 0.0123 0.00718
  [3,] 0.0245 0.00697
  [4,] 0.0368 0.00682
  [5,] 0.0491 0.00672
  [6,] 0.0614 0.00667
  [7,] 0.0736 0.00665
  [8,] 0.0859 0.00668
  [9,] 0.0982 0.00674
 [10,] 0.1104 0.00683
 [11,] 0.1227 0.00695
 [12,] 0.1350 0.00708
 [13,] 0.1473 0.00722
```

```
[14,] 0.1595 0.00737
[15,] 0.1718 0.00752
[16,] 0.1841 0.00767
[17,] 0.1963 0.00780
[18,] 0.2086 0.00792
[19,] 0.2209 0.00803
[20,] 0.2332 0.00811
[21,] 0.2454 0.00817
[22,] 0.2577 0.00821
[23,] 0.2700 0.00822
[24,] 0.2823 0.00821
[25,] 0.2945 0.00818
[26,] 0.3068 0.00813
[27,] 0.3191 0.00806
[28,] 0.3313 0.00799
[29,] 0.3436 0.00790
[30,] 0.3559 0.00782
[31,] 0.3682 0.00774
[32,] 0.3804 0.00767
[33,] 0.3927 0.00763
[34,] 0.4050 0.00761
[35,] 0.4172 0.00762
[36,] 0.4295 0.00767
[37,] 0.4418 0.00777
[38,] 0.4541 0.00793
[39,] 0.4663 0.00814
[40,] 0.4786 0.00842
[41,] 0.4909 0.00876
[42,] 0.5031 0.00918
[43,] 0.5154 0.00967
[44,] 0.5277 0.01024
[45,] 0.5400 0.01089
[46,] 0.5522 0.01160
[47,] 0.5645 0.01239
[48,] 0.5768 0.01325
[49,] 0.5890 0.01417
[50,] 0.6013 0.01515
[51,] 0.6136 0.01617
[52,] 0.6259 0.01725
[53,] 0.6381 0.01835
[54,] 0.6504 0.01948
[55,] 0.6627 0.02063
[56,] 0.6750 0.02178
[57,] 0.6872 0.02293
[58,] 0.6995 0.02406
```

```
 [59,] 0.7118 0.02518
 [60,] 0.7240 0.02626
 [61,] 0.7363 0.02731
 [62,] 0.7486 0.02832
 [63,] 0.7609 0.02927
 [64,] 0.7731 0.03017
 [65,] 0.7854 0.03101
 [66,] 0.7977 0.03179
 [67,] 0.8099 0.03251
 [68,] 0.8222 0.03317
 [69,] 0.8345 0.03377
 [70,] 0.8468 0.03430
 [71,] 0.8590 0.03478
 [72,] 0.8713 0.03520
 [73,] 0.8836 0.03556
 [74,] 0.8958 0.03587
 [75,] 0.9081 0.03614
 [76,] 0.9204 0.03636
 [77,] 0.9327 0.03653
 [78,] 0.9449 0.03667
 [79,] 0.9572 0.03678
 [80,] 0.9695 0.03685
 [81,] 0.9817 0.03690
 [82,] 0.9940 0.03692
 [83,] 1.0063 0.03693
 [84,] 1.0186 0.03692
 [85,] 1.0308 0.03691
 [86,] 1.0431 0.03690
 [87,] 1.0554 0.03690
 [88,] 1.0677 0.03691
 [89,] 1.0799 0.03695
 [90,] 1.0922 0.03703
 [91,] 1.1045 0.03717
 [92,] 1.1167 0.03736
 [93,] 1.1290 0.03764
 [94,] 1.1413 0.03802
 [95,] 1.1536 0.03850
 [96,] 1.1658 0.03912
 [97,] 1.1781 0.03989
 [98,] 1.1904 0.04082
 [99,] 1.2026 0.04194
[100,] 1.2149 0.04327
[101,] 1.2272 0.04482
[102,] 1.2395 0.04661
[103,] 1.2517 0.04865
```

```
[104,] 1.2640 0.05097
[105,] 1.2763 0.05356
[106,] 1.2885 0.05645
[107,] 1.3008 0.05963
[108,] 1.3131 0.06312
[109,] 1.3254 0.06692
[110,] 1.3376 0.07101
[111,] 1.3499 0.07540
[112,] 1.3622 0.08008
[113,] 1.3744 0.08504
[114,] 1.3867 0.09025
[115,] 1.3990 0.09571
[116,] 1.4113 0.10138
[117,] 1.4235 0.10726
[118,] 1.4358 0.11331
[119,] 1.4481 0.11951
[120,] 1.4603 0.12583
[121,] 1.4726 0.13225
[122,] 1.4849 0.13875
[123,] 1.4972 0.14531
[124,] 1.5094 0.15190
[125,] 1.5217 0.15851
[126,] 1.5340 0.16512
[127,] 1.5463 0.17174
[128,] 1.5585 0.17834
[129,] 1.5708 0.18494
[130,] 1.5831 0.19153
[131,] 1.5953 0.19812
[132,] 1.6076 0.20472
[133,] 1.6199 0.21134
[134,] 1.6322 0.21799
[135,] 1.6444 0.22468
[136,] 1.6567 0.23145
[137,] 1.6690 0.23830
[138,] 1.6812 0.24524
[139,] 1.6935 0.25230
[140,] 1.7058 0.25950
[141,] 1.7181 0.26685
[142,] 1.7303 0.27436
[143,] 1.7426 0.28204
[144,] 1.7549 0.28991
[145,] 1.7671 0.29798
[146,] 1.7794 0.30625
[147,] 1.7917 0.31473
[148,] 1.8040 0.32342
```

```
[149,] 1.8162 0.33231
[150,] 1.8285 0.34141
[151,] 1.8408 0.35071
[152,] 1.8530 0.36019
[153,] 1.8653 0.36982
[154,] 1.8776 0.37960
[155,] 1.8899 0.38948
[156,] 1.9021 0.39943
[157,] 1.9144 0.40940
[158,] 1.9267 0.41935
[159,] 1.9390 0.42921
[160,] 1.9512 0.43892
[161,] 1.9635 0.44841
[162,] 1.9758 0.45762
[163,] 1.9880 0.46646
[164,] 2.0003 0.47487
[165,] 2.0126 0.48276
[166,] 2.0249 0.49008
[167,] 2.0371 0.49675
[168,] 2.0494 0.50272
[169,] 2.0617 0.50793
[170,] 2.0739 0.51236
[171,] 2.0862 0.51597
[172,] 2.0985 0.51875
[173,] 2.1108 0.52069
[174,] 2.1230 0.52182
[175,] 2.1353 0.52214
[176,] 2.1476 0.52169
[177,] 2.1598 0.52052
[178,] 2.1721 0.51867
[179,] 2.1844 0.51622
[180,] 2.1967 0.51321
[181,] 2.2089 0.50972
[182,] 2.2212 0.50581
[183,] 2.2335 0.50156
[184,] 2.2457 0.49702
[185,] 2.2580 0.49226
[186,] 2.2703 0.48733
[187,] 2.2826 0.48229
[188,] 2.2948 0.47719
[189,] 2.3071 0.47206
[190,] 2.3194 0.46693
[191,] 2.3317 0.46185
[192,] 2.3439 0.45682
[193,] 2.3562 0.45188
```

```
[194,] 2.3685 0.44703
[195,] 2.3807 0.44228
[196,] 2.3930 0.43764
[197,] 2.4053 0.43312
[198,] 2.4176 0.42870
[199,] 2.4298 0.42439
[200,] 2.4421 0.42017
[201,] 2.4544 0.41604
[202,] 2.4666 0.41198
[203,] 2.4789 0.40797
[204,] 2.4912 0.40400
[205,] 2.5035 0.40003
[206,] 2.5157 0.39606
[207,] 2.5280 0.39205
[208,] 2.5403 0.38797
[209,] 2.5525 0.38381
[210,] 2.5648 0.37953
[211,] 2.5771 0.37511
[212,] 2.5894 0.37053
[213,] 2.6016 0.36578
[214,] 2.6139 0.36085
[215,] 2.6262 0.35572
[216,] 2.6384 0.35039
[217,] 2.6507 0.34487
[218,] 2.6630 0.33916
[219,] 2.6753 0.33328
[220,] 2.6875 0.32725
[221,] 2.6998 0.32109
[222,] 2.7121 0.31483
[223,] 2.7243 0.30849
[224,] 2.7366 0.30210
[225,] 2.7489 0.29571
[226,] 2.7612 0.28932
[227,] 2.7734 0.28299
[228,] 2.7857 0.27673
[229,] 2.7980 0.27056
[230,] 2.8103 0.26452
[231,] 2.8225 0.25863
[232,] 2.8348 0.25289
[233,] 2.8471 0.24733
[234,] 2.8593 0.24195
[235,] 2.8716 0.23676
[236,] 2.8839 0.23176
[237,] 2.8962 0.22696
[238,] 2.9084 0.22236
```

```
[239,] 2.9207 0.21794
[240,] 2.9330 0.21371
[241,] 2.9452 0.20965
[242,] 2.9575 0.20577
[243,] 2.9698 0.20204
[244,] 2.9821 0.19846
[245,] 2.9943 0.19501
[246,] 3.0066 0.19169
[247,] 3.0189 0.18847
[248,] 3.0311 0.18533
[249,] 3.0434 0.18228
[250,] 3.0557 0.17928
[251,] 3.0680 0.17632
[252,] 3.0802 0.17340
[253,] 3.0925 0.17049
[254,] 3.1048 0.16758
[255,] 3.1170 0.16467
[256,] 3.1293 0.16174
[257,] 3.1416 0.15880
[258,] 3.1539 0.15583
[259,] 3.1661 0.15284
[260,] 3.1784 0.14984
[261,] 3.1907 0.14683
[262,] 3.2030 0.14381
[263,] 3.2152 0.14081
[264,] 3.2275 0.13785
[265,] 3.2398 0.13492
[266,] 3.2520 0.13207
[267,] 3.2643 0.12930
[268,] 3.2766 0.12664
[269,] 3.2889 0.12411
[270,] 3.3011 0.12174
[271,] 3.3134 0.11953
[272,] 3.3257 0.11751
[273,] 3.3379 0.11569
[274,] 3.3502 0.11410
[275,] 3.3625 0.11274
[276,] 3.3748 0.11162
[277,] 3.3870 0.11074
[278,] 3.3993 0.11011
[279,] 3.4116 0.10974
[280,] 3.4238 0.10960
[281,] 3.4361 0.10970
[282,] 3.4484 0.11002
[283,] 3.4607 0.11055
```

```
[284,] 3.4729 0.11128
[285,] 3.4852 0.11217
[286,] 3.4975 0.11322
[287,] 3.5097 0.11438
[288,] 3.5220 0.11564
[289,] 3.5343 0.11696
[290,] 3.5466 0.11832
[291,] 3.5588 0.11968
[292,] 3.5711 0.12102
[293,] 3.5834 0.12231
[294,] 3.5957 0.12352
[295,] 3.6079 0.12462
[296,] 3.6202 0.12560
[297,] 3.6325 0.12644
[298,] 3.6447 0.12712
[299,] 3.6570 0.12764
[300,] 3.6693 0.12798
[301,] 3.6816 0.12816
[302,] 3.6938 0.12817
[303,] 3.7061 0.12802
[304,] 3.7184 0.12773
[305,] 3.7306 0.12732
[306,] 3.7429 0.12680
[307,] 3.7552 0.12621
[308,] 3.7675 0.12557
[309,] 3.7797 0.12490
[310,] 3.7920 0.12425
[311,] 3.8043 0.12363
[312,] 3.8165 0.12309
[313,] 3.8288 0.12265
[314,] 3.8411 0.12235
[315,] 3.8534 0.12220
[316,] 3.8656 0.12225
[317,] 3.8779 0.12251
[318,] 3.8902 0.12301
[319,] 3.9024 0.12376
[320,] 3.9147 0.12478
[321,] 3.9270 0.12609
[322,] 3.9393 0.12769
[323,] 3.9515 0.12959
[324,] 3.9638 0.13180
[325,] 3.9761 0.13431
[326,] 3.9884 0.13712
[327,] 4.0006 0.14023
[328,] 4.0129 0.14363
```

```
[329,] 4.0252 0.14731
[330,] 4.0374 0.15125
[331,] 4.0497 0.15545
[332,] 4.0620 0.15989
[333,] 4.0743 0.16454
[334,] 4.0865 0.16940
[335,] 4.0988 0.17444
[336,] 4.1111 0.17966
[337,] 4.1233 0.18502
[338,] 4.1356 0.19052
[339,] 4.1479 0.19614
[340,] 4.1602 0.20186
[341,] 4.1724 0.20766
[342,] 4.1847 0.21354
[343,] 4.1970 0.21947
[344,] 4.2092 0.22544
[345,] 4.2215 0.23143
[346,] 4.2338 0.23742
[347,] 4.2461 0.24340
[348,] 4.2583 0.24933
[349,] 4.2706 0.25519
[350,] 4.2829 0.26096
[351,] 4.2951 0.26659
[352,] 4.3074 0.27206
[353,] 4.3197 0.27733
[354,] 4.3320 0.28236
[355,] 4.3442 0.28711
[356,] 4.3565 0.29154
[357,] 4.3688 0.29560
[358,] 4.3810 0.29926
[359,] 4.3933 0.30248
[360,] 4.4056 0.30524
[361,] 4.4179 0.30749
[362,] 4.4301 0.30923
[363,] 4.4424 0.31043
[364,] 4.4547 0.31108
[365,] 4.4670 0.31119
[366,] 4.4792 0.31075
[367,] 4.4915 0.30979
[368,] 4.5038 0.30831
[369,] 4.5160 0.30635
[370,] 4.5283 0.30392
[371,] 4.5406 0.30107
[372,] 4.5529 0.29783
[373,] 4.5651 0.29424
```

```
[374,] 4.5774 0.29032
[375,] 4.5897 0.28613
[376,] 4.6019 0.28169
[377,] 4.6142 0.27704
[378,] 4.6265 0.27221
[379,] 4.6388 0.26723
[380,] 4.6510 0.26212
[381,] 4.6633 0.25692
[382,] 4.6756 0.25163
[383,] 4.6878 0.24628
[384,] 4.7001 0.24087
[385,] 4.7124 0.23544
[386,] 4.7247 0.22999
[387,] 4.7369 0.22453
[388,] 4.7492 0.21907
[389,] 4.7615 0.21363
[390,] 4.7737 0.20822
[391,] 4.7860 0.20285
[392,] 4.7983 0.19754
[393,] 4.8106 0.19230
[394,] 4.8228 0.18714
[395,] 4.8351 0.18207
[396,] 4.8474 0.17710
[397,] 4.8597 0.17226
[398,] 4.8719 0.16754
[399,] 4.8842 0.16296
[400,] 4.8965 0.15852
[401,] 4.9087 0.15423
[402,] 4.9210 0.15010
[403,] 4.9333 0.14613
[404,] 4.9456 0.14231
[405,] 4.9578 0.13865
[406,] 4.9701 0.13515
[407,] 4.9824 0.13180
[408,] 4.9946 0.12860
[409,] 5.0069 0.12555
[410,] 5.0192 0.12262
[411,] 5.0315 0.11983
[412,] 5.0437 0.11716
[413,] 5.0560 0.11460
[414,] 5.0683 0.11214
[415,] 5.0805 0.10978
[416,] 5.0928 0.10749
[417,] 5.1051 0.10528
[418,] 5.1174 0.10312
```

```
[419,] 5.1296 0.10101
[420,] 5.1419 0.09894
[421,] 5.1542 0.09690
[422,] 5.1664 0.09486
[423,] 5.1787 0.09283
[424,] 5.1910 0.09080
[425,] 5.2033 0.08874
[426,] 5.2155 0.08667
[427,] 5.2278 0.08456
[428,] 5.2401 0.08242
[429,] 5.2524 0.08025
[430,] 5.2646 0.07803
[431,] 5.2769 0.07578
[432,] 5.2892 0.07349
[433,] 5.3014 0.07116
[434,] 5.3137 0.06881
[435,] 5.3260 0.06644
[436,] 5.3383 0.06406
[437,] 5.3505 0.06168
[438,] 5.3628 0.05930
[439,] 5.3751 0.05693
[440,] 5.3873 0.05460
[441,] 5.3996 0.05230
[442,] 5.4119 0.05004
[443,] 5.4242 0.04784
[444,] 5.4364 0.04570
[445,] 5.4487 0.04362
[446,] 5.4610 0.04162
[447,] 5.4732 0.03970
[448,] 5.4855 0.03786
[449,] 5.4978 0.03611
[450,] 5.5101 0.03445
[451,] 5.5223 0.03287
[452,] 5.5346 0.03138
[453,] 5.5469 0.02998
[454,] 5.5591 0.02867
[455,] 5.5714 0.02745
[456,] 5.5837 0.02631
[457,] 5.5960 0.02526
[458,] 5.6082 0.02428
[459,] 5.6205 0.02339
[460,] 5.6328 0.02258
[461,] 5.6450 0.02184
[462,] 5.6573 0.02117
[463,] 5.6696 0.02058
```

```
[464,] 5.6819 0.02005
[465,] 5.6941 0.01960
[466,] 5.7064 0.01920
[467,] 5.7187 0.01887
[468,] 5.7310 0.01860
[469,] 5.7432 0.01838
[470,] 5.7555 0.01822
[471,] 5.7678 0.01811
[472,] 5.7800 0.01804
[473,] 5.7923 0.01802
[474,] 5.8046 0.01803
[475,] 5.8169 0.01808
[476,] 5.8291 0.01815
[477,] 5.8414 0.01825
[478,] 5.8537 0.01836
[479,] 5.8659 0.01848
[480,] 5.8782 0.01861
[481,] 5.8905 0.01873
[482,] 5.9028 0.01884
[483,] 5.9150 0.01894
[484,] 5.9273 0.01902
[485,] 5.9396 0.01907
[486,] 5.9518 0.01908
[487,] 5.9641 0.01906
[488,] 5.9764 0.01899
[489,] 5.9887 0.01887
[490,] 6.0009 0.01870
[491,] 6.0132 0.01848
[492,] 6.0255 0.01821
[493,] 6.0377 0.01788
[494,] 6.0500 0.01750
[495,] 6.0623 0.01707
[496,] 6.0746 0.01660
[497,] 6.0868 0.01608
[498,] 6.0991 0.01553
[499,] 6.1114 0.01494
[500,] 6.1237 0.01433
[501,] 6.1359 0.01370
[502,] 6.1482 0.01307
[503,] 6.1605 0.01243
[504,] 6.1727 0.01180
[505,] 6.1850 0.01118
[506,] 6.1973 0.01058
[507,] 6.2096 0.01000
[508,] 6.2218 0.00947
```

```
[509,] 6.2341 0.00897
[510,] 6.2464 0.00851
[511,] 6.2586 0.00810
[512,] 6.2709 0.00774
[513,] 6.2832 0.00743

Slot "act":
  act
0.305
```

```
result_clo <- fitact(sol_times$clock)      # Activity fitted to clock time
result_clo                                 # Print the original clock time
```

```
An object of class "actmod"
Slot "data":
  [1] 2.094 4.451 2.094 2.094 4.451 4.974 4.189 3.665 2.094 2.356 2.356 6.021
 [13] 3.927 4.189 1.047 3.927 2.094 4.712 4.712 2.094 1.833 3.142 2.880 2.356
 [25] 4.189 3.665 4.189 2.356 4.189 3.927 3.403 2.356 2.356 1.833 1.833 2.618
 [37] 4.712 1.833 4.189 2.356 4.451 4.974 4.712 1.571 2.094 2.094 2.356 3.142
 [49] 1.571 1.833 3.142 3.665 4.189 4.451 2.094 2.094 4.712 2.880 4.189 4.974
 [61] 5.498 2.094 2.356 1.047 2.880 1.833 4.451 2.094 2.618 4.451 2.094 2.356
 [73] 4.712 4.712 1.571 2.094 1.833 2.618 4.189 3.665 4.451 2.094 2.618 2.880
 [85] 3.142 2.618 3.927 2.356 4.712 3.665 2.094 2.880 3.142 1.833 4.712 2.618
 [97] 1.571 2.094 4.189 3.403 2.618 3.142 4.451 4.712 3.927 2.618 2.880 2.356
[109] 2.094 2.618 0.785 3.665 4.451 2.356 2.618 1.571 1.571 1.571 3.403 2.094
[121] 3.142 4.451 4.451 2.618 4.451 2.618 1.571 4.451 4.451 1.571 2.356 6.021
[133] 2.618 2.094 2.356 4.451 3.665 3.142 4.189 3.142 2.880 3.403 2.094 1.833
[145] 1.833 3.665 1.833 1.833 1.833 4.189 2.880 1.833 2.880 1.309 5.236 5.236
[157] 4.712 2.094 4.451 2.094 2.618 5.236 2.094 2.618 2.356 4.189 2.618 4.451
[169] 5.760 4.189 2.880 1.833 2.094 4.974 3.142 4.451 2.094 3.665 4.712 4.712
[181] 5.498 2.356 4.451 2.094 3.927 2.618 4.974 4.451 2.356 4.712 2.356 2.356
[193] 4.712 4.451 4.451 2.094 4.189 5.236 1.571 4.451 2.094 2.094 2.356 2.356
[205] 2.356 2.094 2.356 1.833 2.880 3.142 3.403 1.833 2.356 2.356 2.094 2.094
[217] 2.880 2.094 2.094 4.974 3.927 2.618 4.451 4.712 5.236 4.451 2.880 2.094
[229] 2.356 2.618 2.356 2.618 2.880 2.094 3.665 2.618 3.142 1.309 4.712 4.451
[241] 3.142 2.094 1.833 2.618 5.236 1.571 1.571 1.571 4.974 1.047 1.571 4.451
[253] 4.712 1.833 2.356 4.974 1.833 1.833 0.785 1.571 2.094 2.094 2.356 1.833
[265] 4.451 2.094 3.403 5.236 2.094 3.665 1.833 2.094 2.094 2.618 0.785 4.189
[277] 4.974 2.356 2.618 2.618 2.618 2.356 2.880 3.665 2.356 2.094 2.880 2.094
[289] 1.833 2.618 2.618 2.094 4.451 2.618 2.618 2.094 0.262

Slot "wt":
[1] 1
```

```
Slot "bw":
[1] 34

Slot "adj":
[1] 1

Slot "pdf":
           x       y
  [1,] 0.0000 0.00743
  [2,] 0.0123 0.00718
  [3,] 0.0245 0.00697
  [4,] 0.0368 0.00682
  [5,] 0.0491 0.00672
  [6,] 0.0614 0.00667
  [7,] 0.0736 0.00665
  [8,] 0.0859 0.00668
  [9,] 0.0982 0.00674
 [10,] 0.1104 0.00683
 [11,] 0.1227 0.00695
 [12,] 0.1350 0.00708
 [13,] 0.1473 0.00722
 [14,] 0.1595 0.00737
 [15,] 0.1718 0.00752
 [16,] 0.1841 0.00767
 [17,] 0.1963 0.00780
 [18,] 0.2086 0.00792
 [19,] 0.2209 0.00803
 [20,] 0.2332 0.00811
 [21,] 0.2454 0.00817
 [22,] 0.2577 0.00821
 [23,] 0.2700 0.00822
 [24,] 0.2823 0.00821
 [25,] 0.2945 0.00818
 [26,] 0.3068 0.00813
 [27,] 0.3191 0.00806
 [28,] 0.3313 0.00799
 [29,] 0.3436 0.00790
 [30,] 0.3559 0.00782
 [31,] 0.3682 0.00774
 [32,] 0.3804 0.00767
 [33,] 0.3927 0.00763
 [34,] 0.4050 0.00761
 [35,] 0.4172 0.00762
 [36,] 0.4295 0.00767
 [37,] 0.4418 0.00777
```

```
[38,] 0.4541 0.00793
[39,] 0.4663 0.00814
[40,] 0.4786 0.00842
[41,] 0.4909 0.00876
[42,] 0.5031 0.00918
[43,] 0.5154 0.00967
[44,] 0.5277 0.01024
[45,] 0.5400 0.01089
[46,] 0.5522 0.01160
[47,] 0.5645 0.01239
[48,] 0.5768 0.01325
[49,] 0.5890 0.01417
[50,] 0.6013 0.01515
[51,] 0.6136 0.01617
[52,] 0.6259 0.01725
[53,] 0.6381 0.01835
[54,] 0.6504 0.01948
[55,] 0.6627 0.02063
[56,] 0.6750 0.02178
[57,] 0.6872 0.02293
[58,] 0.6995 0.02406
[59,] 0.7118 0.02518
[60,] 0.7240 0.02626
[61,] 0.7363 0.02731
[62,] 0.7486 0.02832
[63,] 0.7609 0.02927
[64,] 0.7731 0.03017
[65,] 0.7854 0.03101
[66,] 0.7977 0.03179
[67,] 0.8099 0.03251
[68,] 0.8222 0.03317
[69,] 0.8345 0.03377
[70,] 0.8468 0.03430
[71,] 0.8590 0.03478
[72,] 0.8713 0.03520
[73,] 0.8836 0.03556
[74,] 0.8958 0.03587
[75,] 0.9081 0.03614
[76,] 0.9204 0.03636
[77,] 0.9327 0.03653
[78,] 0.9449 0.03667
[79,] 0.9572 0.03678
[80,] 0.9695 0.03685
[81,] 0.9817 0.03690
[82,] 0.9940 0.03692
```

```
 [83,] 1.0063 0.03693
 [84,] 1.0186 0.03692
 [85,] 1.0308 0.03691
 [86,] 1.0431 0.03690
 [87,] 1.0554 0.03690
 [88,] 1.0677 0.03691
 [89,] 1.0799 0.03695
 [90,] 1.0922 0.03703
 [91,] 1.1045 0.03717
 [92,] 1.1167 0.03736
 [93,] 1.1290 0.03764
 [94,] 1.1413 0.03802
 [95,] 1.1536 0.03850
 [96,] 1.1658 0.03912
 [97,] 1.1781 0.03989
 [98,] 1.1904 0.04082
 [99,] 1.2026 0.04194
[100,] 1.2149 0.04327
[101,] 1.2272 0.04482
[102,] 1.2395 0.04661
[103,] 1.2517 0.04865
[104,] 1.2640 0.05097
[105,] 1.2763 0.05356
[106,] 1.2885 0.05645
[107,] 1.3008 0.05963
[108,] 1.3131 0.06312
[109,] 1.3254 0.06692
[110,] 1.3376 0.07101
[111,] 1.3499 0.07540
[112,] 1.3622 0.08008
[113,] 1.3744 0.08504
[114,] 1.3867 0.09025
[115,] 1.3990 0.09571
[116,] 1.4113 0.10138
[117,] 1.4235 0.10726
[118,] 1.4358 0.11331
[119,] 1.4481 0.11951
[120,] 1.4603 0.12583
[121,] 1.4726 0.13225
[122,] 1.4849 0.13875
[123,] 1.4972 0.14531
[124,] 1.5094 0.15190
[125,] 1.5217 0.15851
[126,] 1.5340 0.16512
[127,] 1.5463 0.17174
```

```
[128,] 1.5585 0.17834
[129,] 1.5708 0.18494
[130,] 1.5831 0.19153
[131,] 1.5953 0.19812
[132,] 1.6076 0.20472
[133,] 1.6199 0.21134
[134,] 1.6322 0.21799
[135,] 1.6444 0.22468
[136,] 1.6567 0.23145
[137,] 1.6690 0.23830
[138,] 1.6812 0.24524
[139,] 1.6935 0.25230
[140,] 1.7058 0.25950
[141,] 1.7181 0.26685
[142,] 1.7303 0.27436
[143,] 1.7426 0.28204
[144,] 1.7549 0.28991
[145,] 1.7671 0.29798
[146,] 1.7794 0.30625
[147,] 1.7917 0.31473
[148,] 1.8040 0.32342
[149,] 1.8162 0.33231
[150,] 1.8285 0.34141
[151,] 1.8408 0.35071
[152,] 1.8530 0.36019
[153,] 1.8653 0.36982
[154,] 1.8776 0.37960
[155,] 1.8899 0.38948
[156,] 1.9021 0.39943
[157,] 1.9144 0.40940
[158,] 1.9267 0.41935
[159,] 1.9390 0.42921
[160,] 1.9512 0.43892
[161,] 1.9635 0.44841
[162,] 1.9758 0.45762
[163,] 1.9880 0.46646
[164,] 2.0003 0.47487
[165,] 2.0126 0.48276
[166,] 2.0249 0.49008
[167,] 2.0371 0.49675
[168,] 2.0494 0.50272
[169,] 2.0617 0.50793
[170,] 2.0739 0.51236
[171,] 2.0862 0.51597
[172,] 2.0985 0.51875
```

```
[173,] 2.1108 0.52069
[174,] 2.1230 0.52182
[175,] 2.1353 0.52214
[176,] 2.1476 0.52169
[177,] 2.1598 0.52052
[178,] 2.1721 0.51867
[179,] 2.1844 0.51622
[180,] 2.1967 0.51321
[181,] 2.2089 0.50972
[182,] 2.2212 0.50581
[183,] 2.2335 0.50156
[184,] 2.2457 0.49702
[185,] 2.2580 0.49226
[186,] 2.2703 0.48733
[187,] 2.2826 0.48229
[188,] 2.2948 0.47719
[189,] 2.3071 0.47206
[190,] 2.3194 0.46693
[191,] 2.3317 0.46185
[192,] 2.3439 0.45682
[193,] 2.3562 0.45188
[194,] 2.3685 0.44703
[195,] 2.3807 0.44228
[196,] 2.3930 0.43764
[197,] 2.4053 0.43312
[198,] 2.4176 0.42870
[199,] 2.4298 0.42439
[200,] 2.4421 0.42017
[201,] 2.4544 0.41604
[202,] 2.4666 0.41198
[203,] 2.4789 0.40797
[204,] 2.4912 0.40400
[205,] 2.5035 0.40003
[206,] 2.5157 0.39606
[207,] 2.5280 0.39205
[208,] 2.5403 0.38797
[209,] 2.5525 0.38381
[210,] 2.5648 0.37953
[211,] 2.5771 0.37511
[212,] 2.5894 0.37053
[213,] 2.6016 0.36578
[214,] 2.6139 0.36085
[215,] 2.6262 0.35572
[216,] 2.6384 0.35039
[217,] 2.6507 0.34487
```

```
[218,] 2.6630 0.33916
[219,] 2.6753 0.33328
[220,] 2.6875 0.32725
[221,] 2.6998 0.32109
[222,] 2.7121 0.31483
[223,] 2.7243 0.30849
[224,] 2.7366 0.30210
[225,] 2.7489 0.29571
[226,] 2.7612 0.28932
[227,] 2.7734 0.28299
[228,] 2.7857 0.27673
[229,] 2.7980 0.27056
[230,] 2.8103 0.26452
[231,] 2.8225 0.25863
[232,] 2.8348 0.25289
[233,] 2.8471 0.24733
[234,] 2.8593 0.24195
[235,] 2.8716 0.23676
[236,] 2.8839 0.23176
[237,] 2.8962 0.22696
[238,] 2.9084 0.22236
[239,] 2.9207 0.21794
[240,] 2.9330 0.21371
[241,] 2.9452 0.20965
[242,] 2.9575 0.20577
[243,] 2.9698 0.20204
[244,] 2.9821 0.19846
[245,] 2.9943 0.19501
[246,] 3.0066 0.19169
[247,] 3.0189 0.18847
[248,] 3.0311 0.18533
[249,] 3.0434 0.18228
[250,] 3.0557 0.17928
[251,] 3.0680 0.17632
[252,] 3.0802 0.17340
[253,] 3.0925 0.17049
[254,] 3.1048 0.16758
[255,] 3.1170 0.16467
[256,] 3.1293 0.16174
[257,] 3.1416 0.15880
[258,] 3.1539 0.15583
[259,] 3.1661 0.15284
[260,] 3.1784 0.14984
[261,] 3.1907 0.14683
[262,] 3.2030 0.14381
```

```
[263,] 3.2152 0.14081
[264,] 3.2275 0.13785
[265,] 3.2398 0.13492
[266,] 3.2520 0.13207
[267,] 3.2643 0.12930
[268,] 3.2766 0.12664
[269,] 3.2889 0.12411
[270,] 3.3011 0.12174
[271,] 3.3134 0.11953
[272,] 3.3257 0.11751
[273,] 3.3379 0.11569
[274,] 3.3502 0.11410
[275,] 3.3625 0.11274
[276,] 3.3748 0.11162
[277,] 3.3870 0.11074
[278,] 3.3993 0.11011
[279,] 3.4116 0.10974
[280,] 3.4238 0.10960
[281,] 3.4361 0.10970
[282,] 3.4484 0.11002
[283,] 3.4607 0.11055
[284,] 3.4729 0.11128
[285,] 3.4852 0.11217
[286,] 3.4975 0.11322
[287,] 3.5097 0.11438
[288,] 3.5220 0.11564
[289,] 3.5343 0.11696
[290,] 3.5466 0.11832
[291,] 3.5588 0.11968
[292,] 3.5711 0.12102
[293,] 3.5834 0.12231
[294,] 3.5957 0.12352
[295,] 3.6079 0.12462
[296,] 3.6202 0.12560
[297,] 3.6325 0.12644
[298,] 3.6447 0.12712
[299,] 3.6570 0.12764
[300,] 3.6693 0.12798
[301,] 3.6816 0.12816
[302,] 3.6938 0.12817
[303,] 3.7061 0.12802
[304,] 3.7184 0.12773
[305,] 3.7306 0.12732
[306,] 3.7429 0.12680
[307,] 3.7552 0.12621
```

```
[308,] 3.7675 0.12557
[309,] 3.7797 0.12490
[310,] 3.7920 0.12425
[311,] 3.8043 0.12363
[312,] 3.8165 0.12309
[313,] 3.8288 0.12265
[314,] 3.8411 0.12235
[315,] 3.8534 0.12220
[316,] 3.8656 0.12225
[317,] 3.8779 0.12251
[318,] 3.8902 0.12301
[319,] 3.9024 0.12376
[320,] 3.9147 0.12478
[321,] 3.9270 0.12609
[322,] 3.9393 0.12769
[323,] 3.9515 0.12959
[324,] 3.9638 0.13180
[325,] 3.9761 0.13431
[326,] 3.9884 0.13712
[327,] 4.0006 0.14023
[328,] 4.0129 0.14363
[329,] 4.0252 0.14731
[330,] 4.0374 0.15125
[331,] 4.0497 0.15545
[332,] 4.0620 0.15989
[333,] 4.0743 0.16454
[334,] 4.0865 0.16940
[335,] 4.0988 0.17444
[336,] 4.1111 0.17966
[337,] 4.1233 0.18502
[338,] 4.1356 0.19052
[339,] 4.1479 0.19614
[340,] 4.1602 0.20186
[341,] 4.1724 0.20766
[342,] 4.1847 0.21354
[343,] 4.1970 0.21947
[344,] 4.2092 0.22544
[345,] 4.2215 0.23143
[346,] 4.2338 0.23742
[347,] 4.2461 0.24340
[348,] 4.2583 0.24933
[349,] 4.2706 0.25519
[350,] 4.2829 0.26096
[351,] 4.2951 0.26659
[352,] 4.3074 0.27206
```

```
[353,] 4.3197 0.27733
[354,] 4.3320 0.28236
[355,] 4.3442 0.28711
[356,] 4.3565 0.29154
[357,] 4.3688 0.29560
[358,] 4.3810 0.29926
[359,] 4.3933 0.30248
[360,] 4.4056 0.30524
[361,] 4.4179 0.30749
[362,] 4.4301 0.30923
[363,] 4.4424 0.31043
[364,] 4.4547 0.31108
[365,] 4.4670 0.31119
[366,] 4.4792 0.31075
[367,] 4.4915 0.30979
[368,] 4.5038 0.30831
[369,] 4.5160 0.30635
[370,] 4.5283 0.30392
[371,] 4.5406 0.30107
[372,] 4.5529 0.29783
[373,] 4.5651 0.29424
[374,] 4.5774 0.29032
[375,] 4.5897 0.28613
[376,] 4.6019 0.28169
[377,] 4.6142 0.27704
[378,] 4.6265 0.27221
[379,] 4.6388 0.26723
[380,] 4.6510 0.26212
[381,] 4.6633 0.25692
[382,] 4.6756 0.25163
[383,] 4.6878 0.24628
[384,] 4.7001 0.24087
[385,] 4.7124 0.23544
[386,] 4.7247 0.22999
[387,] 4.7369 0.22453
[388,] 4.7492 0.21907
[389,] 4.7615 0.21363
[390,] 4.7737 0.20822
[391,] 4.7860 0.20285
[392,] 4.7983 0.19754
[393,] 4.8106 0.19230
[394,] 4.8228 0.18714
[395,] 4.8351 0.18207
[396,] 4.8474 0.17710
[397,] 4.8597 0.17226
```

```
[398,] 4.8719 0.16754
[399,] 4.8842 0.16296
[400,] 4.8965 0.15852
[401,] 4.9087 0.15423
[402,] 4.9210 0.15010
[403,] 4.9333 0.14613
[404,] 4.9456 0.14231
[405,] 4.9578 0.13865
[406,] 4.9701 0.13515
[407,] 4.9824 0.13180
[408,] 4.9946 0.12860
[409,] 5.0069 0.12555
[410,] 5.0192 0.12262
[411,] 5.0315 0.11983
[412,] 5.0437 0.11716
[413,] 5.0560 0.11460
[414,] 5.0683 0.11214
[415,] 5.0805 0.10978
[416,] 5.0928 0.10749
[417,] 5.1051 0.10528
[418,] 5.1174 0.10312
[419,] 5.1296 0.10101
[420,] 5.1419 0.09894
[421,] 5.1542 0.09690
[422,] 5.1664 0.09486
[423,] 5.1787 0.09283
[424,] 5.1910 0.09080
[425,] 5.2033 0.08874
[426,] 5.2155 0.08667
[427,] 5.2278 0.08456
[428,] 5.2401 0.08242
[429,] 5.2524 0.08025
[430,] 5.2646 0.07803
[431,] 5.2769 0.07578
[432,] 5.2892 0.07349
[433,] 5.3014 0.07116
[434,] 5.3137 0.06881
[435,] 5.3260 0.06644
[436,] 5.3383 0.06406
[437,] 5.3505 0.06168
[438,] 5.3628 0.05930
[439,] 5.3751 0.05693
[440,] 5.3873 0.05460
[441,] 5.3996 0.05230
[442,] 5.4119 0.05004
```

```
[443,] 5.4242 0.04784
[444,] 5.4364 0.04570
[445,] 5.4487 0.04362
[446,] 5.4610 0.04162
[447,] 5.4732 0.03970
[448,] 5.4855 0.03786
[449,] 5.4978 0.03611
[450,] 5.5101 0.03445
[451,] 5.5223 0.03287
[452,] 5.5346 0.03138
[453,] 5.5469 0.02998
[454,] 5.5591 0.02867
[455,] 5.5714 0.02745
[456,] 5.5837 0.02631
[457,] 5.5960 0.02526
[458,] 5.6082 0.02428
[459,] 5.6205 0.02339
[460,] 5.6328 0.02258
[461,] 5.6450 0.02184
[462,] 5.6573 0.02117
[463,] 5.6696 0.02058
[464,] 5.6819 0.02005
[465,] 5.6941 0.01960
[466,] 5.7064 0.01920
[467,] 5.7187 0.01887
[468,] 5.7310 0.01860
[469,] 5.7432 0.01838
[470,] 5.7555 0.01822
[471,] 5.7678 0.01811
[472,] 5.7800 0.01804
[473,] 5.7923 0.01802
[474,] 5.8046 0.01803
[475,] 5.8169 0.01808
[476,] 5.8291 0.01815
[477,] 5.8414 0.01825
[478,] 5.8537 0.01836
[479,] 5.8659 0.01848
[480,] 5.8782 0.01861
[481,] 5.8905 0.01873
[482,] 5.9028 0.01884
[483,] 5.9150 0.01894
[484,] 5.9273 0.01902
[485,] 5.9396 0.01907
[486,] 5.9518 0.01908
[487,] 5.9641 0.01906
```

```
[488,] 5.9764 0.01899
[489,] 5.9887 0.01887
[490,] 6.0009 0.01870
[491,] 6.0132 0.01848
[492,] 6.0255 0.01821
[493,] 6.0377 0.01788
[494,] 6.0500 0.01750
[495,] 6.0623 0.01707
[496,] 6.0746 0.01660
[497,] 6.0868 0.01608
[498,] 6.0991 0.01553
[499,] 6.1114 0.01494
[500,] 6.1237 0.01433
[501,] 6.1359 0.01370
[502,] 6.1482 0.01307
[503,] 6.1605 0.01243
[504,] 6.1727 0.01180
[505,] 6.1850 0.01118
[506,] 6.1973 0.01058
[507,] 6.2096 0.01000
[508,] 6.2218 0.00947
[509,] 6.2341 0.00897
[510,] 6.2464 0.00851
[511,] 6.2586 0.00810
[512,] 6.2709 0.00774
[513,] 6.2832 0.00743

Slot "act":
  act
0.305
```

```r
# The activity level did not change whether clock time or solar time was used..

# A few more plot options
plot(result_pangolin, data = "none")  # Plot based on our original file with clock time conv
plot(result_clo,
     yunit = "density", add=TRUE, data="n", tline=list(col="cyan"))  # Plot with new clock t
```

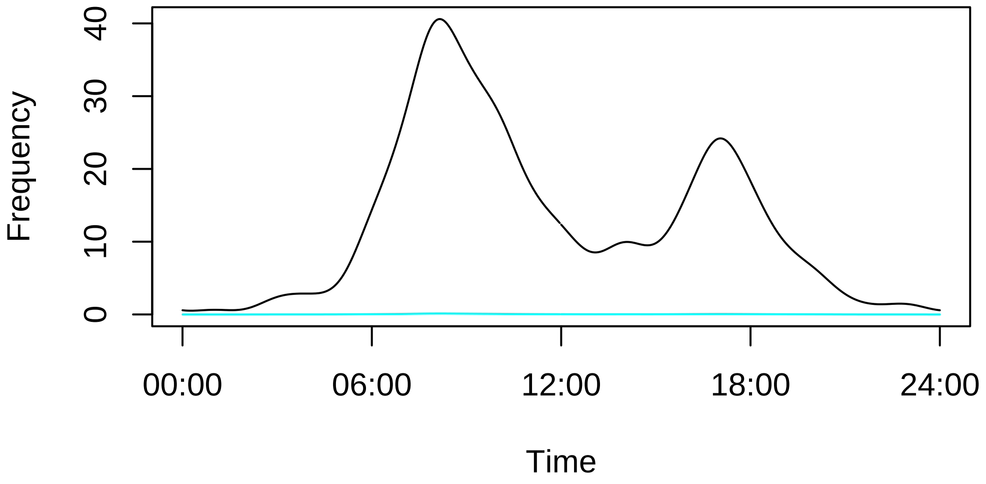


```r
# Lets try that with different time formats
plot(result_pangolin,
     yunit = "density",
     data = "none",
     # ylim = c(0, 1.9),
     )   # Original clock time
abline(v = c(07,19), col = c('black', 'black'), lwd = 2, lty = 'dashed') #abline intended to

plot(result_clo,
     yunit = "density",
     add=TRUE,
     data="n",
     main="clock time ",
     tline=list(col="blue"))   # Clock time from "solartime" covers/matches the original tim
abline(v = c(07,19), col = c('black', 'black'), lwd = 2, lty = 'dashed')

plot(result_sol,
     yunit = "density",
     add=TRUE,
     data="n",
     main="solar time",
     tline=list(col="red"))    # anchored solar time matches the other time types seeingly p
abline(v = c(07,19), col = c('black', 'black'), lwd = 2, lty = 'dashed')
```

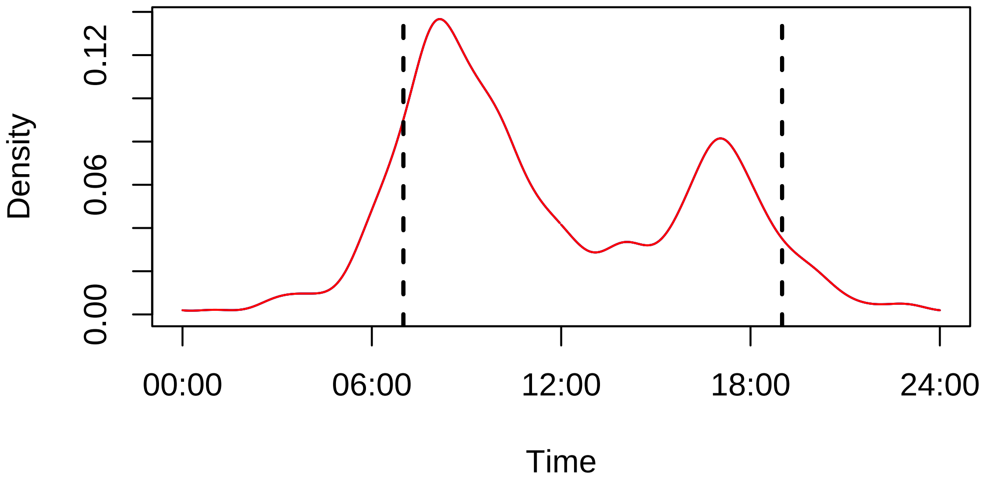


```
# Plot activity pattern, confidence limits, and the sunrise/sunset indicators
plot(result_pangolin_CI,
     yunit = "density",
     cline=list(col="red", lty=3),
     data = "none")
abline(v = c(07,19), col = c('black', 'black'), lwd = 2, lty = 'dashed')
```

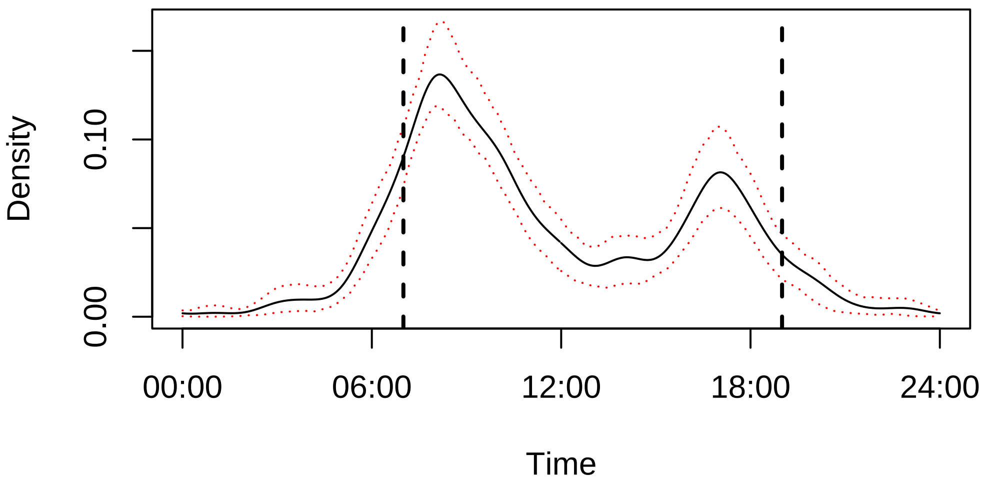

End
