# Supplementary material for: Local Ecological Knowledge Insights Into the Distribution and Activity Patterns of Temminck's Pangolin in Ruaha Landscape, Tanzania
Source: Ecol Evol. 2025 Aug 16;15(8):e71987. doi: 10.1002/ece3.71987 (PMC12357171; doi:10.1002/ece3.71987)
Supplement: Supplementary file 1 — Appendix S1: ece371987‐sup‐0001‐AppendixS1.zip. [file ECE3-15-e71987-s001.zip › Supporting Information.docx]

**Supplemental materials**

Table S1: Number of participants interviewed and their associated ethnic group

| Ethnic number | Ethnic name | Number of participants |
| --- | --- | --- |
| 1 | Barabaig | 24 |
| 2 | Bena | 32 |
| 3 | Fipa | 1 |
| 4 | Gogo | 31 |
| 5 | Hehe | 199 |
| 6 | Kinga | 1 |
| 7 | Lugulu | 1 |
| 8 | Maasai | 30 |
| 9 | Makuwa | 1 |
| 10 | Matego | 1 |
| 11 | Mbulu | 1 |
| 12 | Ndengereko | 4 |
| 13 | Ngoni | 1 |
| 14 | Nyamwezi | 5 |
| 15 | Nyiha | 2 |
| 16 | Safwa | 1 |
| 17 | Sangu | 40 |
| 18 | Sukuma | 11 |
| 19 | Tusi | 1 |
